# Oncogenic mutant KRAS inhibition through oxidation at cysteine 118

**DOI:** 10.1002/1878-0261.13798

**Published:** 2025-01-21

**Authors:** Maximilian Kramer‐Drauberg, Ettore Petrini, Alessia Mira, Enrico Patrucco, Rossella Scardaci, Ilenia Savinelli, Haiyun Wang, Keying Qiao, Giovanna Carrà, Marie‐Julie Nokin, Zhiwei Zhou, Kenneth D. Westover, David Santamaria, Paolo E. Porporato, Chiara Ambrogio

**Affiliations:** ^1^ Department of Molecular Biotechnology and Health Sciences, Molecular Biotechnology Center University of Torino Italy; ^2^ School of Life Sciences and Technology Tongji University Shanghai China; ^3^ Department of Clinical and Biological Sciences University of Torino Orbassano Italy; ^4^ Laboratory of Tumor and Development Biology, GIGA‐Cancer University of Liege Belgium; ^5^ Department of Biochemistry The University of Texas Southwestern Medical Center Dallas TX USA; ^6^ Department of Radiation Oncology The University of Texas Southwestern Medical Center Dallas TX USA; ^7^ Molecular Mechanisms of Cancer Program, Centro de Investigación del Cáncer CSIC‐Universidad de Salamanca Spain

**Keywords:** cysteine modification, KRAS C118, NSCLC, oncogene, redox regulation, ROS

## Abstract

Specific reactive oxygen species activate the GTPase Kirsten rat sarcoma virus (KRAS) by reacting with cysteine 118 (C118), leading to an electron transfer between C118 and nucleoside guanosine diphosphate (GDP), which causes the release of GDP. Here, we have mimicked permanent oxidation of human KRAS at C118 by replacing C118 with aspartic acid (C118D) in KRAS to show that oncogenic mutant KRAS is selectively inhibited via oxidation at C118, both *in vitro* and *in vivo*. Moreover, the combined treatment of hydrogen‐peroxide‐producing pro‐oxidant paraquat and nitric‐oxide‐producing inhibitor *N*(ω)‐nitro‐l‐arginine methyl ester selectively inhibits human mutant KRAS activity by inducing oxidization at C118. Our study shows for the first time the vulnerability of human mutant KRAS to oxidation, thereby paving the way to explore oxidation‐based anti‐KRAS treatments in humans.

## Graphical abstract

Oncogenic mutant Kirsten rat sarcoma virus (KRAS) is one of the major drivers of human cancer, making it a key target in the fight against the disease. This study discovered a novel mechanism to selectively inhibit oncogenic human mutant KRAS through oxidation at cysteine 118. This finding opens new avenues for exploring potential oxidation‐based anti‐KRAS treatments in humans. The PyMOL Molecular Graphics System, Version 3.0.4, Schrödinger, LLC has been used to generate KRAS model.

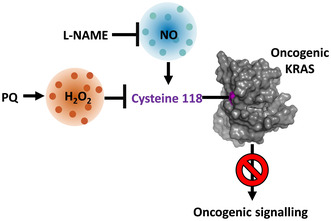

**Abbreviations**
4OHT: 4‐hydroxytamoxifen
AKT: protein‐kinase B
C118: cysteine 118
CRISPR: clustered regularly interspaced short palindromic repeats
DMEM: Dulbecco's modified Eagle medium
ERK: extracellular signal‐regulated kinases
FBS: fetal bovine serum
GDP: nucleoside guanosine diphosphate
GTP: nucleotide guanosine triphosphate
H2DCFDA: 2′,7′‐dichlorodihydrofluorescein diacetate dye
H_2_O_2_
: hydroxy peroxide
HA‐tag: human influenza hemagglutinin
HSP90: heat shock protein 90
KRAS: Kirsten rat sarcoma virus
KRASmut: KRAS oncogenic mutant
L‐Name: *N*(ω)‐nitro‐l‐arginine methyl ester
MEFs: mouse embryonic fibroblasts
MitoSox: MitoSOX superoxide indicators
mTOR: mechanistic target of rapamycin
NAC: *N*‐acetyl cysteine
NO: nitric oxide
NOS: nitric oxide synthases
PEG‐PCMAL: Polyethylene Glycol‐Photo Cleavable Maleimide
PI3K: phosphoinositide 3‐kinases
PQ: paraquat
ROS: reactive oxygen species
S6: ribosomal protein S6
SOD: superoxide dismutase

## 1 Introduction

One of the main drivers of human cancer is mutant RAS. The human genome contains three closely related *RAS* genes: *KRAS*, *NRAS*, and *HRAS* [1]. Approximately one in seven cancers contains a mutation at codon 12, 13, or 61 in the *KRAS* gene, which renders the protein predominantly nucleotide guanosine triphosphate (GTP) bound and hence active and oncogenic [2, 3, 4, 5]. The most common oncogenic Kirsten rat sarcoma virus (KRAS) mutations in order of frequency are G12D, G12V, and G12C [6]. The inhibition of specific KRAS mutants is now possible through the recent development of KRAS G12C and G12D mutant specific inhibitors along with panRAS inhibitors [7, 8, 9]. Despite encouraging preclinical outcomes, clinical trials have illuminated a pattern of initial response followed by the emergence of resistance in patients treated with KRAS inhibitors [10, 11, 12]. To drive malignant transformation and tumorigenesis, oncogenic KRAS regulates a plethora of processes including the context dependent production or reduction of reactive oxygen species (ROS) [13]. ROS are chemically reactive molecules containing oxygen that have the potential to damage all biological macromolecules, yet they can also function as signaling molecules by introducing distinct post‐translational modifications to specific redox‐sensitive target proteins [14]. As part of the mutant KRAS‐mediated redox balancing process, mutant KRAS promotes the generation of mitochondrial ROS [15]. The generation of mitochondrial ROS is essential for KRAS‐mediated carcinogenesis [16]. Notably, RAS not only regulates ROS production but is itself regulated by reactive molecules [17]. The free‐radical oxidant nitric oxide (NO) can activate RAS by causing a thiyl radical intermediate at the cysteine thiol residue of the most surface‐accessible and redox‐sensitive cysteine 118 (C118) of nucleoside guanosine diphosphate (GDP)‐bound RAS, which causes the release of GDP and the subsequent replacement by GTP [18, 19, 20, 21]. The end‐product of this reaction is the creation of a covalent bond between NO and the thiol residue of C118, termed S‐nitrosylation [18, 19, 21]. The presence of the C118 S‐nitrosylation prevents RAS from engaging in further radical‐based nucleotide exchanges [22]. Endothelial nitric oxide synthase‐derived NO activates wild‐type RAS via C118 S‐nitrosylation; this stimulation of wild‐type RAS can promote oncogenic mutant RAS‐driven tumorigenesis [23, 24].

In contrast to radical oxidants, the nonradical oxidant hydrogen peroxide (H_2_O_2_) does not facilitate a RAS nucleotide exchange [25]. However, H_2_O_2_ facilitates a RAS GDP dissociation in the presence of transition metals [26], converting H_2_O_2_ into the highly reactive hydroxyl radical (OH^·^) [27]; this confirms that RAS is activated exclusively by free‐radical oxidants. The central role of C118 as KRAS redox sensor has been previously proposed. Various reports indicate that substituting the redox‐sensitive C118 with serine (C118S) renders RAS impervious to activation by free‐radical oxidants, while leaving its biochemical functions unaffected, including the intrinsic and nucleotide exchange factor (GEF)‐mediated guanine nucleotide dissociation rate, the protein structure, the GTPase activity, and the capacity to bind an effector [18, 21, 22, 28, 29, 30, 31, 32, 33, 34]. Besides wild‐type RAS, oncogenic mutant KRAS is also activated via a C118 redox reaction. In mice, the impact of the carcinogen urethane, known to induce Kras mutation‐positive lung tumors, was mitigated by the C118S substitution [35]. Furthermore, the inhibitory effect of the C118S substitution on the radical‐mediated activation of mutant RAS was evident in mouse embryonic fibroblasts (MEFs) expressing KRAS C118S in *cis* with the oncogenic mutation G13D. These cells exhibited reduced activation of the MAPK pathway upon stimulation with EGF compared to the Kras^G13D^ single‐mutant [35]. Congruent with the observation that C118 is a key redox target, previous investigations in *Caenorhabditis elegans* revealed that the ortholog of mutant KRAS is inhibited via oxidation at the conserved C118 by the nonradical oxidant H_2_O_2_ [36]. Mimicking a permanent oxidation by H_2_O_2_ at C118 by the CRISPR‐induced substitution of C118 with aspartic acid (C118D) completely abolished the mutant KRAS ortholog tumor‐like phenotype [36]. Based on this finding, we hypothesized that oxidation at C118 by H_2_O_2_ will inhibit human KRAS. Despite research on the role of C118 in KRAS biology, there is still limited evidence regarding the role of C118 in human KRAS, especially concerning its effects on mutant oncogenic KRAS. In this study, we investigated whether the mechanism of mutant KRAS inhibition through oxidation at C118 is maintained in mammalian cells *in vitro* and *in vivo*, and we explored the therapeutic value of KRAS oxidation.

## 2 Materials and methods

### 2.1 Animal models

For allograft experiments, athymic Nude‐*Foxn1nu+* mice (females, 6–8‐week old) were purchased from Inotiv (former Envigo, Indianapolis, IN, USA). KRas^lox^ KRAS^MUT^ MEFs expressing wild‐type KRAS or mutant KRAS with or without the in *cis* C118S or C118D substitution (2 × 10^6^) were injected subcutaneously in the flanks of recipient mice. Once tumors were detectable, tumor growth measurements were taken every 3 days using a caliper. Mice were euthanized when reached humane endpoint and tumors were resected. Samples of the tumors were snap frozen and the remaining part was fixed in 10% buffered formalin and embedded in paraffin for further analysis.

Mice were kept, managed, and sacrificed at the MBC Animal Facility of the University of Torino, according to current European (2007/526/CE) legislation in accordance with the guideline for Ethical Conduct in the Care and Use of Animals as stated in The International Guiding Principles for Biomedical Research Involving Animal. The mice were housed in cages with adequate space, bedding material for comfort and maintained under specific pathogen free conditions, while maintaining 12‐h dark/light cycle. Ambient temperature was kept within 20–24 °C, and humidity levels ranged from 45% to 65%. All experiments were approved by the Italian Health Minister (authorization no. 1227/2020‐PR).

### 2.2 Cell lines

*HRas* ^*−/−*^; *NRas* ^*−/−*^; *KRas* ^*lox/lox*^ MEFs were generated in Mariano Barbacid's lab [37, 38]. Cells were grown at 37 °C and 5% CO_2_ in a humidified incubator in Dulbecco's modified Eagle medium (DMEM) supplemented with 10% fetal bovine serum, 100 mg·mL^−1^ penicillin, and 100 units·mL^−1^ streptomycin (complete medium). Human cancer cell lines H441 (RRID:CVCL_1561), H2887 (RRID:CVCL_5159), H23 (RRID:CVCL_1547), H358 (RRID:CVCL_1559), A427 (RRID:CVCL_1055), and SK‐LU‐1 (RRID:CVCL_0629) cells were purchased from the ATCC (Manassas, VA, USA) and grown at 37 °C and 5% CO_2_ in a humidified incubator in DMEM medium supplemented with 10% fetal bovine serum (FBS), 100 mg·mL^−1^ penicillin, and 100 units·mL^−1^ streptomycin (complete medium). Cell lines used in the study tested negative for mycoplasma as determined by the Mycoplasma Plus PCR Primer Set (Agilent, Santa Clara, CA, USA). Cell lines were authenticated by Eurofins Genomics (Ebersberg, Germany) using the AmpFLSTR™ Identifiler™ Plus PCR Amplification Kit (Thermo Fisher Scientific, Waltham, MA, USA).

### 2.3 Generation of KRas^lox^ KRAS^MUT^ cells

KRas^lox^ KRAS^MUT^ cells were generated as previously described [39]. In Brief, KRAS^G12C^, KRAS^G12C/C118S^, KRAS^G12C/C118D^, KRAS^G12D^, KRAS^G12D/C118S^, KRAS^G12D/C118D^, KRAS^G12V^, KRAS^G12V/C118S^, and KRAS^G12V/C118D^ retroviral plasmids were created by point mutagenesis from a pBABE HA‐tagged KRAS^WT^ plasmid (a gift from Channing, Addgene plasmid #75282, Watertown, MA, USA) using QuikChange XL Site‐Directed Mutagenesis Kit (Cat #200516; Agilent). Retroviruses were generated by co‐transfection of pBABE plasmids together with pAmpho plasmid into 293T cells using Effectene Transfection Reagent (Cat #301425; Qiagen, Venlo, Netherlands). The retroviruses were transduced into HRas^−/−^; NRas^−/−^; KRas^lox/lox^ MEFs followed by 2 weeks of puromycin selection (1 μg·mL^−1^) in complete DMEM medium. To induce endogenous KRas deletion, cells were then cultured for at least 10 days in the presence of 4‐hydroxytamoxifen (4OHT; Sigma, 600 nmol·L^−1^, Cat #H6278, Darmstadt, Germany). KRas^lox^ KRAS^MUT^ cells are available under a material transfer agreement.

### 2.4 Real‐time quantitative PCR

To assess the human KRAS gene expression level, total RNA was extracted from cells using a standard TRIZOL isolation protocol (Cat #15596018; Thermo Fisher). One microgram of RNA was reverse transcribed with random primers using the RevertAid RT Kit (Cat #00940535; Thermo Fisher) following the manufacturer's instructions. Real‐time PCR was performed on a 7500 Fast Real‐Time PCR System (Applied Biosystems) with the PowerTrack SYBR Green Master Mix (Cat #00864923; Applied Biosystems) according to the manufacturer's instruction. As previously described in Ref. [40], for the quantitative detection of human KRAS transcripts, the following primer sets were used: Human KRAS Fw: GGACTGGGGAGGGCTTTCT and Human KRAS Rev: GCCTGTTTTGTGTCTACTGTTCT. Murine ACTB (beta actin) expression levels were used to normalize for differences in RNA input. Results represent the average of three independent biological samples, each of which was consecutively amplified twice in triplicate.

### 2.5 Growth assessment by IncuCyte

The growth rate of KRas^lox^ KRAS^MUT^ cells MEFs cells was assessed as previously described in Ref. [39]. Cells (1 × 10^3^) were seeded on 96‐well plates in 200 μL DMEM complete medium. For low Serum conditions, cells (3 × 10^3^) were seeded on 96‐well plates in 100 μL DMEM complete medium. After overnight incubation, cells were washed three times with PBS, and 200 μL of DMEM medium supplemented with 0.5% FBS was added to the plates. For the drug assessment, cells (1 × 10^3^) were seeded in 96‐well plates in 100 μL DMEM complete medium. After overnight incubation 100 μL DMEM complete medium containing no drug, paraquat (PQ) alone or in combination with *N*(ω)‐nitro‐l‐arginine methyl ester (L‐NAME), L‐NAME, AMG510, MRTX1133, RMC‐4998, MRTX849, *N*‐acetyl cysteine (NAC) alone or in combination with PQ, and L‐NAME was added to achieve a concentration of 35 μm PQ, 1 mm L‐NAME, 1 μm AMG510, 0.2 μm RMC‐4998, 1 μm MRTX1133, and 4 mm NAC. The plates were then incubated in the IncuCyte S3 for real‐time imaging, with four fields imaged per well under 10× magnification every 2 h for a total of 60–90 h. Data were analyzed using the incucyte 2022A software, which quantified cell surface area coverage as confluence values. IncuCyte experiments were performed in triplicate. A single representative growth curve is shown for each condition. The data were graphically displayed using graphpad prism 8 for Windows (GraphPad Software, Insight Venture Management, LLC, New York City, NY, USA).

### 2.6 Western blot

Western blotting was performed as previously described [41]. Briefly, cells from *in vitro* culture or *ex vivo* explants were lysed in RIPA lysis buffer (Cat #89900; Thermo Fisher) supplemented with Halt protease and phosphatase inhibitor cocktail (Cat #78445; Thermo Fisher). Fifteen microgram of protein extracts were separated by SDS/PAGE (Bio‐Rad, Hercules, CA, USA), transferred to a PVDF membrane, and blotted with primary antibodies raised against RAS (Cat #8832; Cell Signaling Technology, Danvers, MA, USA), HA‐Tag (6E2) (1 : 1000, Cat #2367; Cell Signaling), HSP90 (1 : 1000, Cat #4874; Cell Signaling), Phospho‐AKT (Ser473) (D9E) (1 : 2000, Cat #4060; Cell Signaling), Phospho‐p44/42 MAPK (pErk1/2) (Thr202/Tyr204) (1 : 1000, Cat #9101; Cell Signaling), Phospho‐S6 Ribosomal Protein (Ser240/244) (1 : 1000, Cat #2215; Cell Signaling), Phospho‐AKT (Ser473) (D9E) (1 : 2000, Cat #4060; Cell Signaling), AKT (1 : 1000, Cat #9272; Cell Signaling), p44/42 MAPK (Erk1/2) (1 : 1000, Cat #9102; Cell Signaling), and S6 Ribosomal Protein (5G10) (1 : 1000, Cat #2217; Cell Signaling). Secondary anti‐mouse or anti‐rabbit antibodies include ECL Sheep anti‐Mouse IgG HRP‐linked secondary antibody (Cat #NA931V; GE Healthcare, Chicago, IL, USA) and ECL Donkey anti‐rabbit IgG, and HRP‐linked secondary antibody (Cat #NA934V; GE Healthcare). Western blotting showed in the manuscript are representative of at least three independent experiments.

### 2.7 Quantification of ROS levels

Cells (2 × 10^6^) were seeded in 6‐well plates in complete Media and incubated overnight; for total intracellular ROS measurement cells were also incubated in media containing 35 and 500 μm PQ. The next day, cells were harvested using an Accutase enzyme, washed with an HBSS buffer and incubated with 300 r.p.m. agitation in the dark for 20 min at 37 °C with 5 μm MitoSOX™ (M36008; Invitrogen, Thermo Fisher Scientific) or 5 μm 2′,7′‐dichlorodihydrofluorescein diacetate (DCFH‐DA) (D6883; Sigma‐Aldrich, Burlington, MA, USA). The cells were then washed with an HBSS buffer and the mean fluorescence intensity of oxidized MitoSOX (to detect mitochondrial ROS) or the mean fluorescence intensity of the DCFH‐DA oxidation product 2′‐7′dichlorofluorescein (DCF) (to detect total intracellular ROS) in the samples was assessed using a (BD FACSCelesta™, BD Biosciences, Franklin Lakes, NJ, USA) flow cytometer. Results represent the average of four independent biological samples.

### 2.8 KRAS activation assay

KRAS activation assay was performed as previously described [42]. Briefly, KRAS activity was measured with the active RAS pull‐down and detection kit (Cat #11871; Cell Signaling Technology). Briefly, glutathione *S*‐transferase–RAF1 RAS‐binding domain and glutathione agarose resin were mixed together with whole‐cell lysates and incubated on a rotator for 1 h at 4 °C, followed by three washes and elution with 2× SDS/PAGE loading buffer. The samples were then analyzed by SDS/PAGE and western blot analysis with primary antibodies raised against RAS.

### 2.9 Redox state analysis of KRAS

Cells were seeded in 96‐well plates. After an overnight incubation, cells were treated or not treated for 3 h with complete DMEM containing 1 m PQ. Cells were washed with PBS and then solubilized in RIPA lysis buffer (Cat #89900; Thermo Fisher) containing 0.5 mmol·L^−1^ SulfoBiotics PEG‐PCMAL (Dojindo Europe Gmbh, Munich, Germany), as a control, cells were solubilized in RIPA buffer without PEG‐PCMal. The mixture was incubated for 30 min at 37 °C and then treated with the nonreducing Laemmli buffer, followed by loading onto SDS/PAGE gels (Bio‐Rad). After electrophoresis, the gel was exposed to UV rays for 10 min using the ChemiDoc XRS+ System (Bio‐Rad) to cleave the PEG moiety, after which the proteins were transferred to a PVDF membrane and blotted with primary antibodies raised against RAS.

### 2.10 Synergistic drug effect measurement

Cells (1 × 10^3^) were seeded in 96‐well plates. After an overnight incubation, cells were treated with dactolisib (up to 1 μm) and trametinib (up to 1 μm) alone or in combination for 72 h. The synergy between the two compounds in KRas^lox^KRAS^MUT^ cells was assessed using the Zero Interaction Potency method and the open‐source synergyfinder 3.0 software (https://synergyfinderplus.org).

### 2.11 Statistical analysis

Values are presented as mean ± standard error of the mean unless stated otherwise. graphpad prism 8.0.1 (GraphPad Inc.) software was used for statistical analyses. For comparisons established only between two groups, we used Student's two‐tailed, unpaired *t*‐test. Datasets consisting of more than two groups statistical significance was determined by a one‐way ANOVA using Bonferroni's correction for multiple comparison. Survival analysis was performed using the Kaplan–Meier method and between‐group differences in survival were tested using the Log‐rank (Mantel–Cox) test. IncuCyte experiments, real‐time quantitative PCRs, western blots, and biochemical assays were performed in triplicate. *n* values mentioned in the figure legends indicate the number of animals used per experimental group. A *P*‐value that was < 0.05 was considered statistically significant for all datasets.

### 2.12 Key resources

All reagents and resources used in this paper are provided as Table S1.

## 3 Results

### 3.1 Characterization of Ras‐less MEFs expressing human wild‐type KRAS or mutant KRAS with and without the in *cis* C118S or C118D substitution

**Fig. 1:**
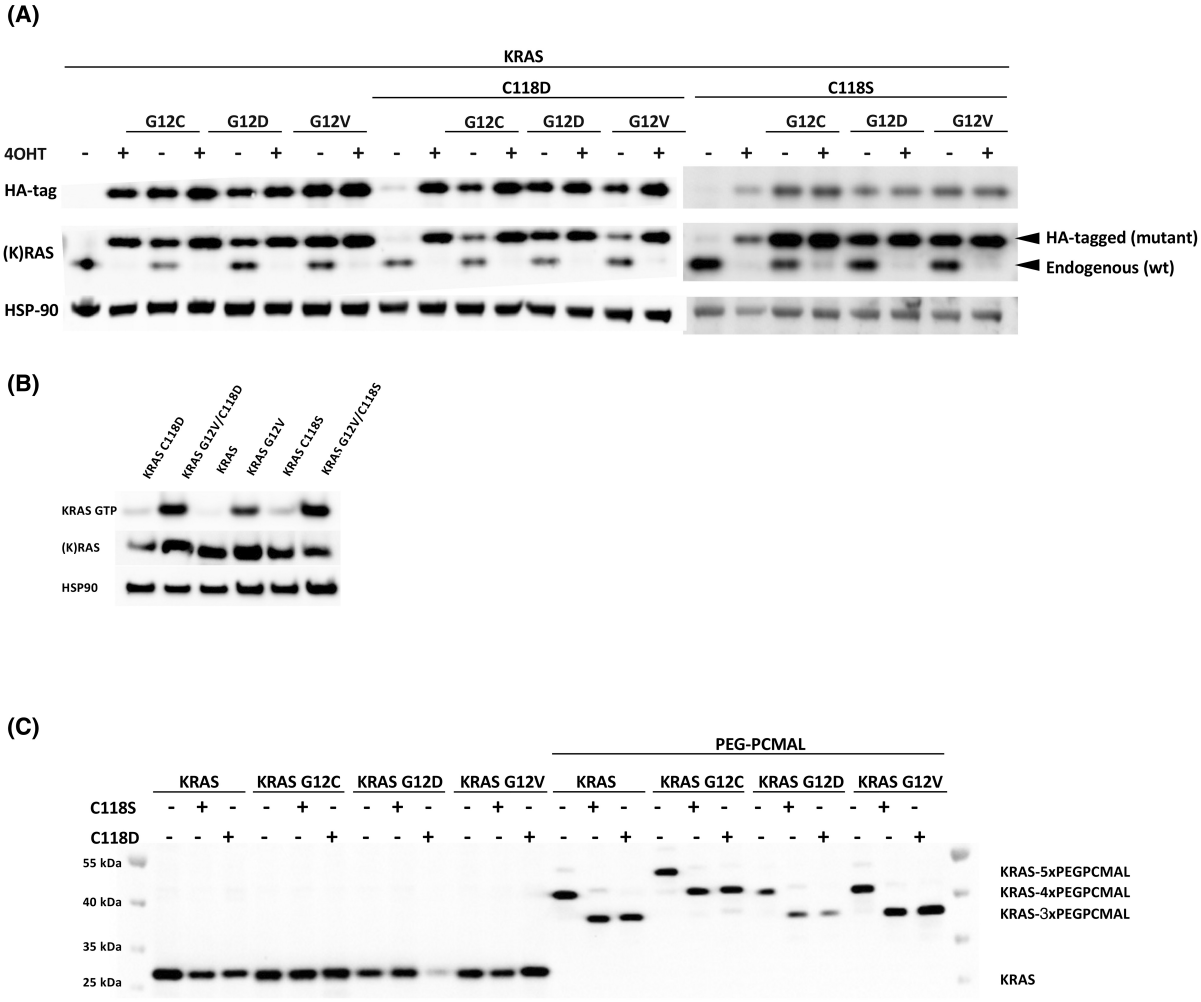
The introduction of either the C118S or C118D substitution renders KRAS redox‐insensitive without compromising its biochemical function or altering the protein levels of KRAS. (A) Hras^−/−^; Nras^−/−^; Kras^lox/lox^ mouse embryonic fibroblasts (MEFs) stably transduced with human influenza hemagglutinin‐tagged (HA‐tagged) human KRAS^WT^ and KRAS^G12C^, KRAS^G12D^ or KRAS^G12V^ mutant with or without the in *cis* C118S or C118D substitution (KRas^lox^ KRAS^MUT^ cells) were cultured in the presence or absence of 4‐hydroxytamoxifen (4OHT) and analyzed by western blot to confirm endogenous KRAS removal and to determine exogenous KRAS expression. Results are representative of one of three similar experiments. (B) Ras‐GTP levels in KRas^lox^ KRAS^MUT^ MEFs expressing KRAS^WT^ or KRAS^G12V^ with or without the in *cis* C118S or C118D substitution. Results are representative of one of three similar experiments. (C) Polyethylene glycol maleimide (PEG‐PC‐Mal) labeling of KRAS cysteine‐thiol residues in KRas^lox^ KRAS^MUT^ MEFs expressing KRAS^WT^ and KRAS^G12V^, KRAS^G12C^, or KRAS^G12D^ with or without the in *cis* C118S or C118D substitution. Results are representative of one of three similar experiments.

To determine the effect of C118 oxidation on mutant KRAS activity, we replaced C118 with aspartic acid in KRAS (C118D) to mimic a permanent oxidation by H_2_O_2_, or with serine (C118S) to inhibit oxidation at C118. These substitutions were introduced either alone or in *cis* with the most common oncogenic mutations in KRAS (G12C/G12D/G12V), using the KRas^lox^ KRAS^MUT^ system [39]. We transduced HRas^−/−^; NRas^−/−^; KRas^lox/lox^ MEFs with the human HA‐tagged wild‐type and with G12V, G12C or G12D mutant KRAS4B (herein referred to KRAS) with and without the in *cis* C118S or C118D substitutions. After abolishing the expression of endogenous wild‐type KRas by treating the cells with 4OHT, we assessed the effect of the C118S and C118D substitutions on wild‐type and mutant human KRAS. We found the protein level (Fig. 1A) and the mRNA expression level (Fig. S1) of wild‐type and mutant KRAS carrying the C118S or C118D substitution were similar to the controls. Moreover, neither the C118S substitution nor the C118D substitution had a significant effect on the ability of wild‐type or mutant KRAS to bind to GTP (Fig. 1B).

To ascertain the redox state of KRAS cysteines across various mutants, we used PEG‐PCMal. PEG‐PCMal selectively binds free cysteine thiol groups, inducing a mobility shift of approximately 5 kDa in the electrophoretic analysis for each thiol group bound. We found that PEG‐PCMal binds all human KRAS cysteines (Fig. 1C). Whereas, when C118 is mutated to either C118S or C118D, PEG‐PCMal binds KRAS one time less resulting in a 5‐kDa mobility shift reduction (Fig. 1C). These data demonstrate the redox insensitivity of KRAS at C118 when C118 is removed through the introduction of both C118S and C118D substitutions.

In addition, we determined that neither the C118S substitution nor the C118D substitution interfere with the inhibiting effect of KRAS G12C or KRAS G12D specific inhibitors (Fig. S2A,B).

According to the literature, aside from making RAS redox‐insensitive, a C118S substitution has no measurable effect on the ability of RAS to bind to effectors, on its protein structure, on its intrinsic and GEF‐mediated guanine nucleotide dissociation rate, or on its GTPase activity [18, 21, 22, 28, 29, 30, 31, 32, 33] which harmonizes with the aforementioned results establishing that the C118S substitution makes KRAS redox‐insensitive without affecting the KRAS protein level, the mRNA level, or the GTP loading (Fig. 1). Furthermore, we found the same to be true for the C118D substitution (Fig. 1).

### 3.2 Mimicking oxidation at C118 by hydrogen peroxide inhibits KRAS mutant signaling activity

**Fig. 2:**
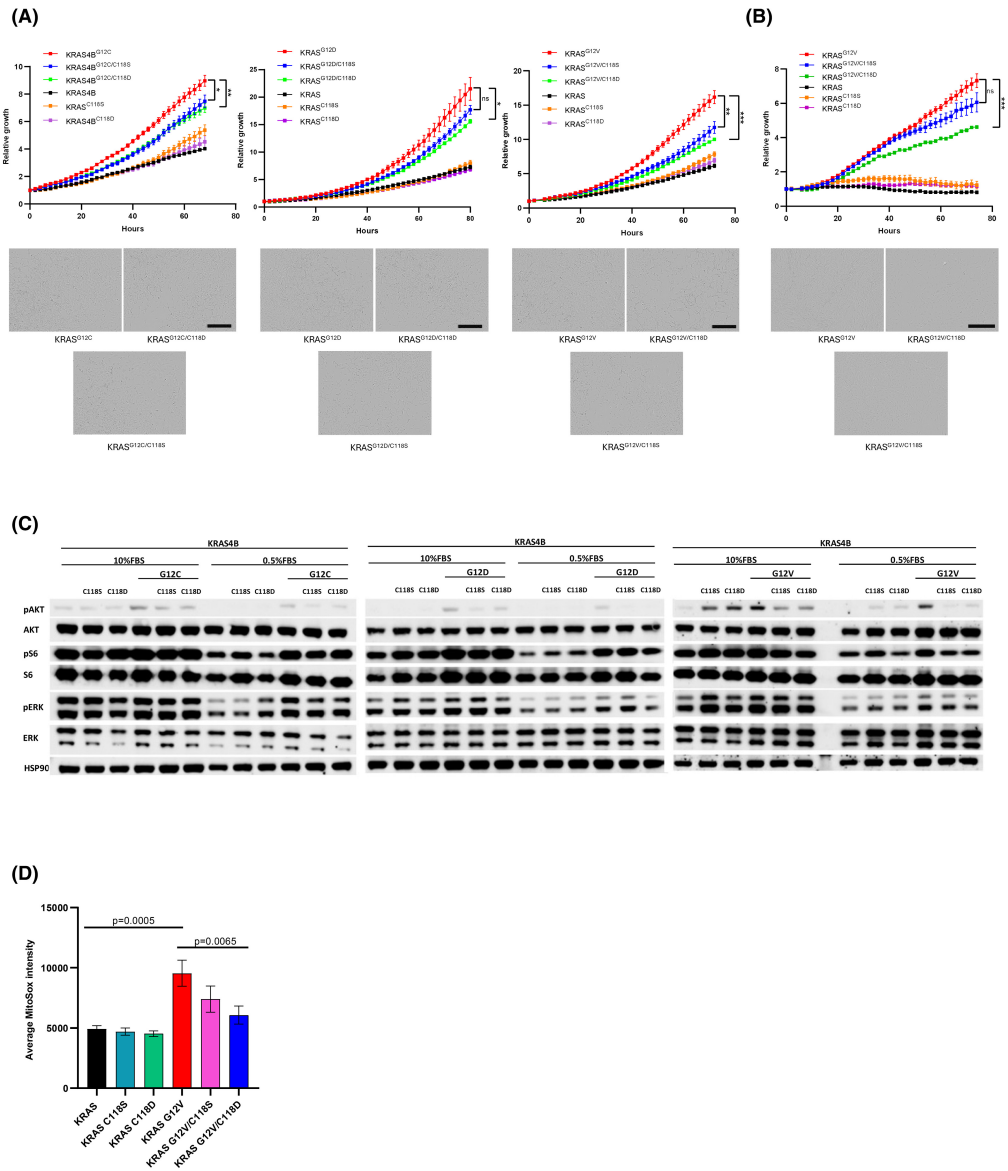
The C118D substitution strongly inhibits KRAS mutant signaling activity, while the C118S substitution exerts a weaker inhibitory effect. (A) Growth rates of in KRas^lox^ KRAS^MUT^ MEFs expressing KRAS^WT^, KRAS^G12C^, KRAS^G12D^ or KRAS^G12V^ with or without the in *cis* C118S or C118D substitution grown in 10% fetal bovine serum (FBS) medium assessed by IncuCyte measurements. Unpaired Student's test was used to evaluate statistical significance between KRAS mutant cells with or without the C118S substitution and between KRAS mutant cells with or without the C118D substitution (ns, not significant; *P < 0.05; **P < 0.01; ***P < 0.001). Results are representative of one of three similar experiments (scale bar: 400 μm), error bars represent mean ± SEM. (B) Growth rates of in KRas^lox^ KRAS^MUT^ MEFs expressing KRAS^WT^ or KRAS^G12V^ with or without the in *cis* C118S or C118D substitution grown in 0.5% FBS medium assessed by IncuCyte measurements. Unpaired Student's test was used to evaluate statistical significance between KRAS G12V cells with or without the C118S substitution and between KRAS G12V cells with or without the C118D substitution (ns, not significant; ***P < 0.001). Results are representative of one of three similar experiments (scale bar: 400 μm), error bars represent mean ± SEM. (C) RAS downstream effector signaling in KRas^lox^ KRAS^MUT^ MEFs expressing KRAS^WT^, KRAS^G12C^, KRAS^G12D^ or KRAS^G12V^ with or without the in *cis* C118S or C118D substitution. Results are representative of one of three similar experiments. (D) Mean mitochondrial Reactive Oxygen Species (ROS) levels detected with MitoSOX in KRas^lox^ KRAS^MUT^ MEFs expressing KRAS^WT^, KRAS^C118S^, KRAS^C118D^, KRAS^G12V^, KRAS^G12V/C118S^, or KRAS^G12V/C118D^. Error bars represent mean ± SEM (*P* = 0.0005 and *P* = 0.0065; one‐way ANOVA test followed by the Bonferroni's multiple comparison test to correct for multiple comparisons).

To scrutinize the impact of oxidation on KRAS signaling activity, we evaluated the growth of KRas^lox^ KRAS^MUT^ MEFs expressing wild‐type or mutant KRAS with or without the in *cis* C118S or C118D substitution. Notably, we observed a pronounced inhibitory effect on the growth of MEFs expressing human KRAS^G12C^, KRAS^G12D^, or KRAS^G12V^ upon introducing either the C118S or C118D substitution (Fig. 2A). Moreover, the extent of growth inhibition caused by the C118D substitution varied in the KRAS mutants: MEFs expressing KRAS^G12V^ demonstrated the most substantial inhibition, followed by a moderate inhibition in MEFs expressing KRAS^G12C^, and a weaker inhibition in MEFs expressing KRAS^G12D^. Conversely, the proliferation of cells with wild‐type KRAS remained unaffected by either the C118S or C118D substitutions. Additionally, under serum‐starvation conditions where the reliance on mutant KRAS for stimulating cell growth is heightened, we observed a robust selective inhibition of mutant KRAS^G12V^‐driven cell growth with the C118D substitution and a milder inhibition with the C118S substitution (Fig. 2B). Moreover, under prolonged 5‐day serum‐starvation conditions, the inhibition of mutant KRAS^G12V^‐driven cell growth by the C118S substitution, and particularly by the C118D substitution, becomes even more pronounced (Fig. S3). This consistency aligns with our findings in 10% serum DMEM.

Subsequently, we examined the impact of C118S and C118D substitutions on the main KRAS downstream effectors in normal and low serum conditions. In the KRAS^WT^ background, neither of the C118 substitutions affected the RAS downstream effector activation (Fig. 2C). In contrast, in MEFs that express mutant KRAS, both C118 substitutions reduced the RAS downstream effector AKT and S6 but had little to no effect on the ERK phosphorylation levels (Fig. 2C). Of note, in 0.5% FBS, the inhibitory effect of the C118S and C118D substitutions on the activation of the KRAS mutant effector was more pronounced than in 10% FBS media (Fig. 2C). Additionally, to further evaluate the contribution of AKT‐S6 and ERK signaling, we treated cells with the corresponding inhibitors dactolisib (PI3Ki) and trametinib (ERKi) alone or in combination, analyzing their synergistic effect (Fig. S4). These results showed that KRas^lox^ KRAS^MUT^ cells expressing KRAS^G12V^ are more sensitive to the treatment than KRAS^G12D^ and KRAS^G12C^ mutant cell lines.

To further investigate the effect of the C118S and C118D substitutions on mutant KRAS activity, we quantified mitochondrial ROS production using the mitochondrial superoxide indicator MitoSOX. In concordance with previous studies [16], cells expressing mutant KRAS^G12V^ exhibited an elevated level of mitochondrial ROS compared to cells expressing wild‐type KRAS. This heightened mitochondrial ROS level in KRAS^G12V^‐expressing cells was mitigated by 46% by the C118S substitution and by 75% by the C118D substitution, approaching levels seen in normal wild‐type cells (Fig. 2D).

Collectively, our results support the notion that blocking the radical‐mediated activation through both the C118S and C118D substitutions effectively inhibits mutant KRAS activity while sparing wild‐type KRAS activity.

### 3.3 The combination treatment of pro‐oxidant PQ and NO‐production inhibitor L‐NAME inhibits mutant KRAS by targeting C118

**Fig. 3:**
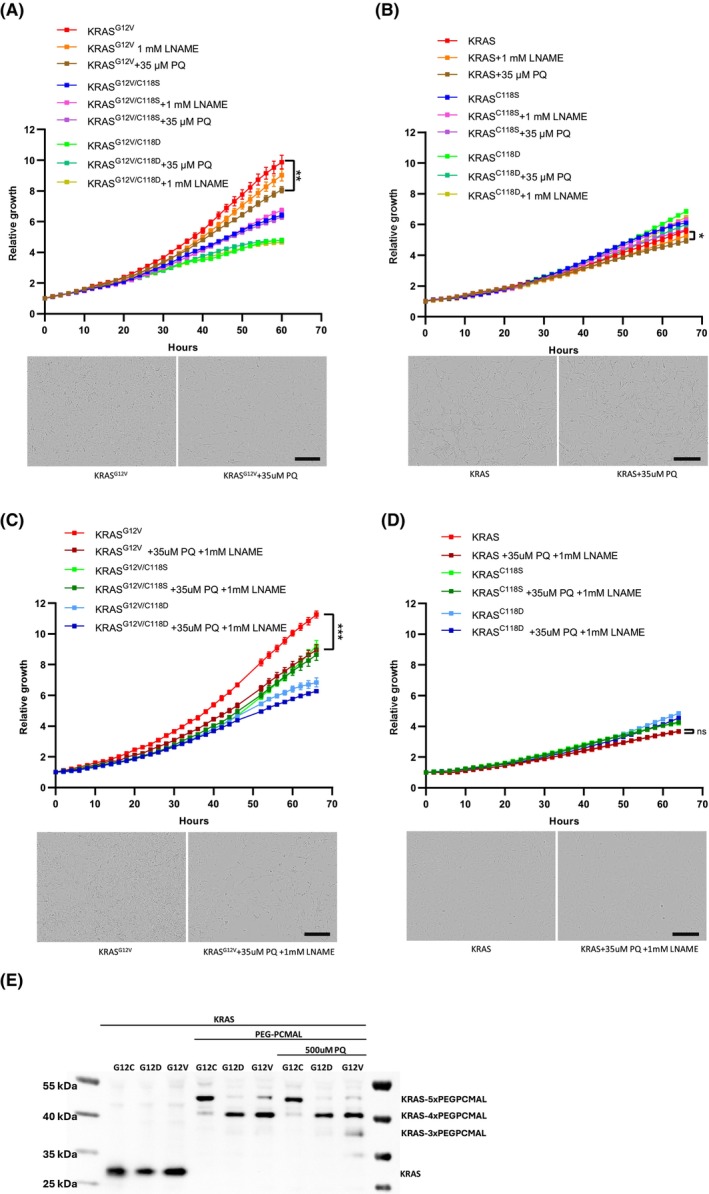
Through inducing oxidation at C118, Paraquat (PQ) inhibits the growth signaling of KRAS^G12V^. (A) Growth rates of KRas^lox^ KRAS^MUT^ mouse embryonic fibroblasts (MEFs) expressing KRAS^G12V^, KRAS^G12V/C118S^, and KRAS^G12V/C118D^ treated or not treated with 35 μm PQ or 1 mm *N*(ω)‐nitro‐l‐arginine methyl ester (L‐NAME) assessed by IncuCyte measurements. Unpaired Student's test was used to evaluate statistical significance between KRAS^G12V^ cells treated or not treated with PQ (**P < 0.01). Results are representative of one of three similar experiments (scale bar: 400 μm), error bars represent mean ± SEM. (B) Growth rates of KRas^lox^ KRAS^MUT^ MEFs expressing KRAS^WT^, KRAS^C118S^, and KRAS^C118D^ treated or not treated with 35 μm PQ or 1 mm L‐NAME assessed by IncuCyte measurements. Unpaired Student's test was used to evaluate statistical significance between KRAS^WT^ cells treated or not treated with PQ (*P < 0.05). Results are representative of one of three similar experiments (scale bar: 400 μm), error bars represent mean ± SEM. (C) Growth rates of KRas^lox^ KRAS^MUT^ MEFs expressing KRAS^G12V^, KRAS^G12V/C118S^, and KRAS^G12V/C118D^ treated or not treated with a combination of 35 μm PQ and 1 mm L‐NAME assessed by IncuCyte measurements. Unpaired Student's test was used to evaluate statistical significance between KRAS^G12V^ cells treated or not treated with the combination of 35 μm PQ and 1 mm L‐NAME (***P < 0.001). Results are representative of one of three similar experiments (scale bar: 400 μm), error bars represent mean ± SEM. (D) Growth rates of KRas^lox^ KRAS^MUT^ MEFs expressing KRAS^WT^, KRAS^C118S^, and KRAS^C118D^ treated or not treated with a combination of 35 μm PQ and 1 mm L‐NAME assessed by IncuCyte measurements. Unpaired Student's test was used to evaluate statistical significance between KRAS^WT^ cells treated or not treated with the combination of 35 μm PQ and 1 mm L‐NAME (ns, not significant). Results are representative of one of three similar experiments (scale bar: 400 μm), error bars represent mean ± SEM. (E) Polyethylene glycol maleimide (PEG‐PC‐Mal) labeling of KRAS cysteine‐thiol residues MEFs expressing KRAS^G12C^, KRAS^G12D^ or KRAS^G12V^ treated or not treated with 500 μm PQ for 3 h. Results are representative of one of three similar experiments.

We recently discovered that H_2_O_2_ produced by pro‐oxidant PQ inhibits the KRAS mutant ortholog in *C. elegans* via a reaction at C118 [36]. PQ generates superoxide which is rapidly converted into H_2_O_2_ by superoxide dismutases (SODs) [43]. To assess the impact of PQ on human KRAS signaling, we treated MEFs expressing wild‐type or mutant KRAS with or without the in *cis* C118S or C118D substitution with 35 μm PQ. We found PQ to selectively inhibit the growth of MEFs expressing KRAS^G12V^ with minimal to no effect on MEFs expressing KRAS^G12V/C118S^, KRAS^G12V/C118D^, KRAS^WT^, KRAS^C118S^, or KRAS^C118D^ (Fig. 3A,B). Notably, C118 is identified as the target of PQ's inhibitory effect, as demonstrated by the observation that both C118S and C118D substitutions, which render KRAS redox‐insensitive, abolish the inhibitory effect of PQ on the KRAS^G12V^ growth signaling activity (Fig. 3A). Furthermore, by detecting the cellular ROS level using the cellular ROS indicator 2′,7′‐dichlorodihydrofluorescein diacetate (H_2_DCFDA), we found that a concentration of 35 μm PQ does not elevate ROS levels in MEFs expressing wild‐type or mutant KRAS^G12V^ (Fig. S5). This suggests that a treatment with 35 μm PQ only minimally increases the ROS level in the appropriate subcellular location to inhibit mutant KRAS, and does not increase the cellular ROS level to a cytotoxic level. Additionally, we tested whether the growth of different human cell lines harboring KRAS^G12V^ (H441, H2887), KRAS^G12C^ (H23, H358), and KRAS^G12D^ (A427, SK‐LU‐1) mutations is affected by PQ treatment. We found that, except for H23, the growth of human cell lines harboring a KRAS^G12V^ mutation was strongly inhibited by PQ treatment, whereas cells harboring KRAS^G12C^ or KRAS^G12D^ mutations were only weakly affected (Fig. S6).

Cysteine C118 in mammalian RAS is a known potential target of NO; a reaction with NO results in the activation of the protein. However, the end product of this reaction, C118 S‐nitrosylation, prevents any further radical‐mediated modifications at C118 [22] and we hypothesize that it also inhibits H_2_O_2_‐mediated modifications. Consequently, we propose that by inhibiting NOS and thus NO‐production, we could potentially enhance the reaction of C118 with H_2_O_2_, thereby increasing the inhibition of mutant KRAS. To test this hypothesis, cells were treated with a combination of NOS inhibitor L‐NAME and PQ. Remarkably, the combination of 1 mm L‐NAME and 35 μm PQ selectively inhibits the growth of MEFS that express KRAS^G12V^ to a greater degree than the PQ treatment alone and has little to no effect on MEFs that express KRAS^G12V/C118S^, KRAS^G12V/C118D^, KRAS^WT^, KRAS^C118S^, or KRAS^C118D^ (Fig. 3C,D). The degree of KRAS^G12V^ inhibition by the combination treatment closely resembles the effect produced by mimicking oxidation at C118 through the C118D substitution (Fig. 2A). Consistent with our previous finding showing the inhibition of mutant KRAS driven growth via the removal of C118 through the C118 substitutions (Fig. 2A), treatment with L‐NAME alone slightly inhibits the growth of MEFs expressing KRAS^G12V^ and not MEFs expressing KRAS^G12V/C118S^, KRAS^G12V/C118D^, KRAS^WT^, KRAS^C118S^, or KRAS^C118D^ (Fig. 3A,B). This suggests that KRAS^G12V^ is normally partially activated by C118 S‐nitrosylation. Together these observations indicate that the inhibition of KRAS^G12V^ by PQ is enhanced when NO production by NOS is inhibited.

Furthermore, in line with a previous study revealing that a treatment with the anti‐oxidant NAC enhances the growth of cells driven by mutant KRAS [44], we found that NAC increases the growth of MEF expressing KRAS^G12V^ (Fig. S7). Notably, this growth‐promoting effect of NAC is independent of the C118 substitutions and of the PQ and L‐NAME combination (Fig. S7). These findings suggest that, within our experimental system, NAC does not effectively diminish the specific subcellular pool of ROS responsible for inhibiting mutant KRAS through C118 oxidation.

To ascertain whether PQ inhibits mutant KRAS by directly oxidizing C118, we examined the cysteine oxidation status of KRAS using the reagent PEG‐PCMal in MEFs expressing KRAS^G12C^, KRAS^G12D^, or KRAS^G12V^ treated or untreated with a high level of PQ for a brief period. In untreated conditions, PEG‐PCMAL binds all KRAS cysteines resulting in an electrophoresis shift of KRAS (a 25‐kDa shift in the case of KRAS^G12D^ or KRAS^G12V^, and a 30 kDa shift in the case of KRAS^G12C^, which possess one additional cysteine compared to the other mutants) (Fig. 3E). In contrast, in cells expressing KRAS^G12V^ but not in cells expressing KRAS^G12C^ or KRAS^G12D^ treated with PQ, PEG‐PCMAL binding to KRAS is partially inhibited for one cysteine, as indicated by the appearance of another KRAS band with a 20 kDa mobility shift. These results indicate that the PQ treatment induces a partial oxidation of one of the cysteines, which we assume to be C118 since it is the most surface exposed cysteine of KRAS, making it unavailable to bind with PEG PCMAL. In summary, PQ appears to act directly on KRAS^G12V^ activity by causing cysteine oxidation, particularly at C118.

### 3.4 Mimicking oxidation at C118 through the C118D substitution inhibits mutant KRAS driven tumor growth

**Fig. 4:**
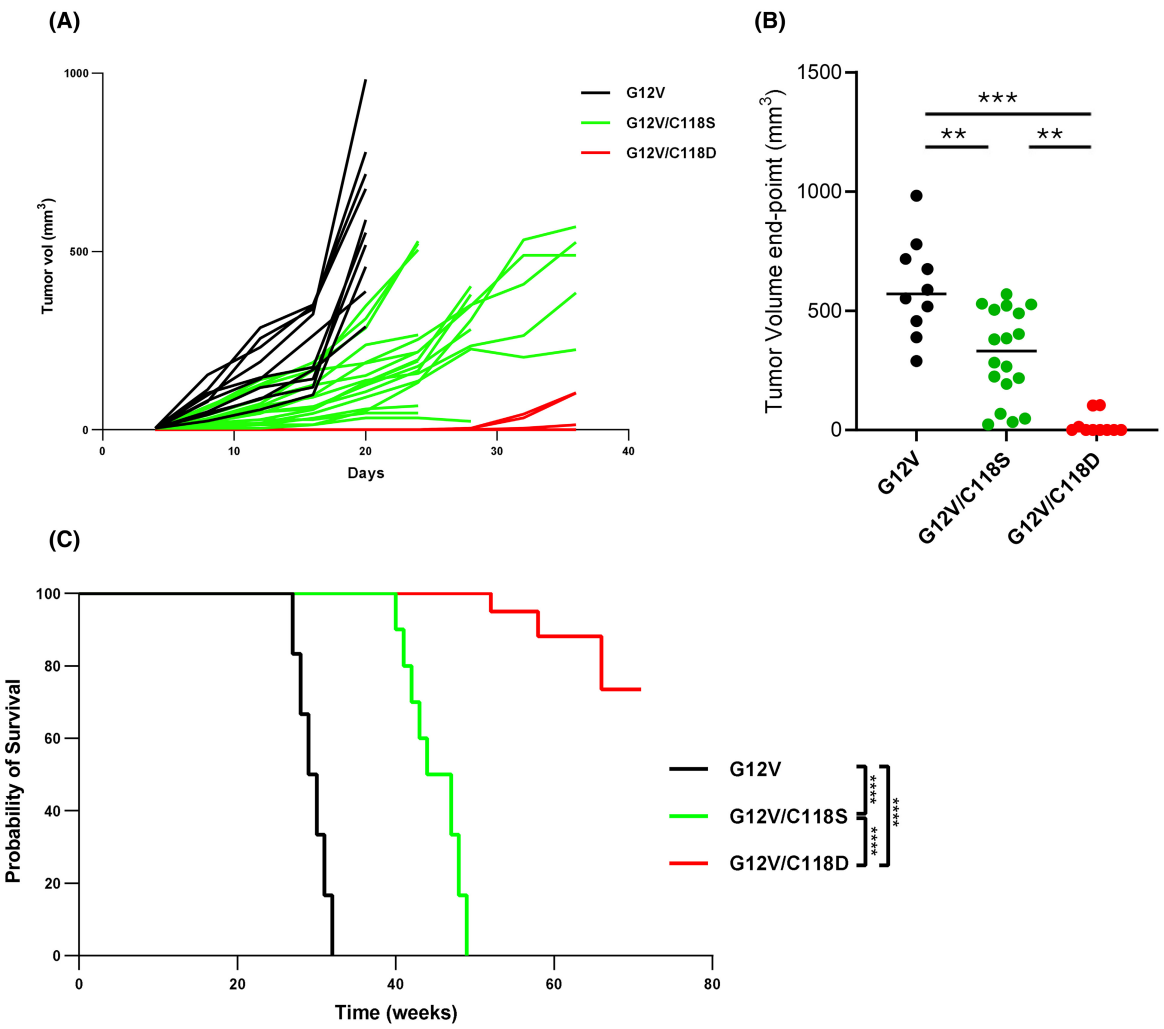
*In vivo*, the C118D substitution robustly inhibits KRAS mutant signaling activity, whereas the C118S substitution exerts a weaker inhibitory effect. (A) KRas^lox^ KRAS^MUT^ mouse embryonic fibroblasts (MEFs) expressing KRAS^G12V^, KRAS^G12V/C118S^, or KRAS^G12V/C118D^ were injected subcutaneously into nude mice. Tumor growth was followed over time by measuring tumor volume every 3 days with a caliper, KRAS^G12V^ (*n* = 10), KRAS^G12V/C118S^ (*n* = 18) or KRAS^G12V/C118D^ (*n* = 16). (B) Tumor growth measure at endpoint in KRAS^G12V^, KRAS^G12V/C118S^, or KRAS^G12V/C118D^ sub‐cutis tumors (**P < 0.01, ***P < 0.005, one‐way ANOVA Kruskal–Wallis test). (C) Kaplan–Meier analysis of mice injected subcutaneously with KRas^lox^ KRAS^MUT^ MEFs expressing KRAS^G12V^ (*n* = 6, black line), KRAS^G12V/C118S^ (*n* = 8, turquoise line), or KRAS^G12V/C118D^ (*n* = 8, red line) (****P < 0.0001; log‐rank test [Mantel–Cox]).

Our findings establish a means to inhibit KRAS^G12V^ activity by hindering the oxidation of KRAS at C118 through the C118S substitution and notably by mimicking the oxidation of KRAS at C118 through the C118D substitution. To assess the impact of the C118 substitutions on oncogenic KRAS signaling *in vivo*, we subcutaneously implanted KRas^lox^ KRAS^MUT^ MEFs expressing KRAS^G12V^, KRAS^G12V/C118S^, or KRAS^G12V/C118D^ into mice and assessed their *in vivo* tumor growth and survival rate. We observed that the ability of MEFs expressing KRAS^G12V^ to form tumors was constrained by both C118 substitutions (Fig. 4A,B), although KRAS^G12V/C118S^ eventually grew and reached an average tumor volume comparable to the control KRAS^G12V^ cell. On the contrary, the C118D substitution exhibited a definite stronger effect, which is consistent with our *in vitro* data. These results were mirrored by the increased survival rates of mice bearing KRAS^G12V/C118S^ or KRAS^G12V/C118D^ tumors as compared to the controls. The short survival rate of mice implanted with MEFs expressing KRAS^G12V^ was extended by the C118S substitution and even further prolonged by the C118D substitution (Fig. 4C). Subsequently, we determined the RAS effector activation in *ex‐vivo* tumor samples taken at the end point of the survival experiment. We found ERK and Akt activation to be heterogeneous, with no clear reduction in tumors expressing KRAS^G12V/C118D^ compared to those expressing KRAS^G12V^ or KRAS^G12V/C118S^ (Fig. S8).

Altogether, these results demonstrate that impairing mutant KRAS oxidation *in vivo* could provide an effective therapeutic strategy for KRAS‐driven tumors.

## 4 Discussion

The inhibition of mutant KRAS remains a prime objective in the treatment of human cancers. Here we have demonstrated for the first time that human mutant KRAS is inhibited through oxidation at C118 with the nonradical oxidant H_2_O_2_. By mimicking a permanent C118 oxidation with H_2_O_2_ in KRAS through the introduction of the C118D substitution, which also makes KRAS redox insensitive (Fig. 1C), we can inhibit the increased rate of proliferation of MEFs driven by mutant KRAS (Fig. 2A,B). The inhibitory impact of the C118D substitution on KRAS is selective to mutant KRAS as the proliferation of MEFs expressing wild‐type KRAS is not inhibited by the introduction of the C118D substitution (Fig. 2A,B). Moreover, we observed varying degrees of inhibition among different KRAS mutants (G12C, G12D, and G12V) when harboring the C118D substitution, with KRAS^G12V^ exhibiting the most pronounced inhibition (Fig. 2A). Consistent with this observation, we also found that human cell lines harboring a KRAS^G12V^ mutation were more strongly inhibited by the pro‐oxidant PQ than those containing a KRAS^G12C^ or KRAS^G12D^ mutation, except for the H23 cell line, which we assume is due to other genetic alterations making it PQ sensitive (Fig. S6). This observation suggests that differences in the mutants are linked to events that involve the oxidation of C118.

Based on the data from this study, we can only speculate on the specific mechanisms underlying the differences in behavior between codon 12 mutants. One possible explanation may involve differential solvent exposure of C118 among the mutants, which could impact accessibility to compounds like PQ. Several studies have demonstrated that oncogenic mutations can affect switch 1 and 2 dynamics, potentially leading to variations in the solvent exposure of C118 [45, 46, 47]. Structural analysis suggests that Cys 118 appears to be more solvent‐accessible in configurations where switch 1 is displaced, such as in rapid nucleotide exchange mutants like A146T. However, differences in solvent exposure between G12V and other mutants like G12C and G12D have not been formally assessed. Another possible mechanistic explanation for differential effects of C118 manipulation is related to variations in nucleotide cycling. Biochemical studies have shown that KRAS G12V hydrolyzes GTP at a slower rate than G12C and G12D [45, 46, 47, 48], suggesting that a higher proportion of KRAS G12V may exist in the GTP‐bound state compared to other mutants. This difference could influence RAS–RAS interaction dynamics at the cell membrane, where GTP binding is crucial for RAS signaling and function. This argument is further supported by examining a proposed structural model of biological RAS–RAS interactions, which occur at the α4‐α5 interface [39, 45, 46, 47, 48] This model is backed by the presence of this interaction in crystal contacts within X‐ray structures of KRAS and structure–function analyses, including *in vivo* studies. In that model, C118 is part of the RAS–RAS interaction interface through a water network (Fig. S9), and substitution to C118D alters the electrostatic environment, and would be expected to negatively alter RAS–RAS interaction dynamics according to that model.

**Fig. 5:**
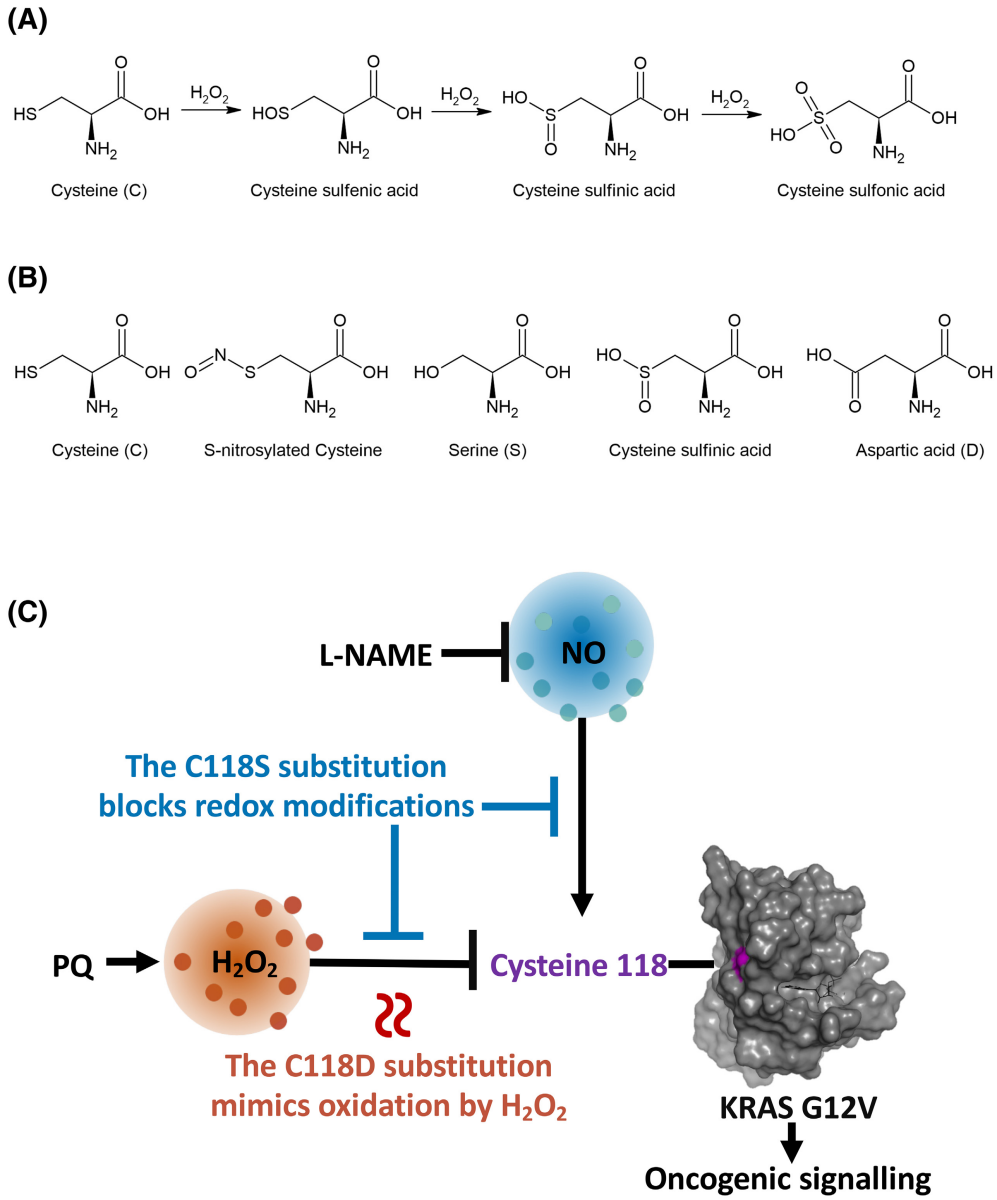
A model depicting the two reactive molecule pathways that regulate KRAS^G12V^ activity by reacting with C118. (A) Oxidative modification of cysteine with hydrogen peroxide (H_2_O_2_). (B) Oxidative modifications of cysteine and mimics of nonoxidizable and oxidized forms. Serine mimics a nonoxidizable cysteine, whereas aspartic acid mimics cysteine sulfinic acid, a doubly oxidized form of cysteine. (C) Mutant KRAS inhibiting redox‐pathway: *in vitro* and *in vivo* H_2_O_2_ selectively inhibits KRAS^G12V^ mutant driven oncogenesis by introducing cysteine 118 (C118) sulfinic acid modification without affecting wild‐type KRAS. The source of H_2_O_2_ can be paraquat, which produces superoxide that is converted by superoxide dismutases (SODs) to H_2_O_2_. The inhibitory effect of H_2_O_2_ oxidation on KRAS^G12V^ activity is permanently mimicked by the C118D substitution which resembles a cysteine sulfinic acid. Mutant activating redox‐pathway: Nitric oxide (NO) activates oncogenic KRAS^G12V^ mutant signaling introducing a C118 S‐nitrosylation. L‐NAME can inhibit the NO‐mediated activation of KRAS by inhibiting the NO production by nitric oxide synthases. Both the KRAS inhibiting and activating pathways are abolished by the C118S substitution, rendering KRAS redox‐insensitive. H_2_O_2_ and NO are depicted as acting on the same KRAS C118, although it is possible that the two reactive molecules act on different KRAS proteins and/or at different times during tumor development.

The oxidation of cysteine by H_2_O_2_ can induce post‐translational modifications which impact a protein's activity [49]. When a cysteine thiolate reacts with H_2_O_2_, a cysteine sulfinic acid is formed and is subjected to several alternative fates, one of which is to further react with H_2_O_2_ to generate a cysteine sulfinic acid and then a cysteine sulfonic acid modification (Fig. 5A) [50]. The C118D substitution replaces C118 with an Aspartic acid. Since Aspartic acid is similar in molecular shape and charge to cysteine sulfinic acid [51], we are able to mimic a permanent cysteine sulfinic acid modification via the C118D substitution (Fig. 5B). Given that the C118D substitution inhibits mutant KRAS activity, we propose that the specific type of oxidative post‐translational modification responsible for inhibiting mutant KRAS is the cysteine sulfinic acid modification. The C118D substitution mimics a permanent cysteine sulfinic acid oxidative modification, which is apt considering the cysteine sulfinic acid modification, with a few exceptions, is generally an irreversible oxidative modification [52].

We determined that the C118D substitution neither affected the protein/mRNA level of KRAS (Fig. 1A and Fig. S1) nor impacted the binding of KRAS to GTP (Fig. 1B) nor influenced the activation of the RAS downstream RAF/MEK/ERK pathway (the C118D substitution only minimally reduced the activation of the RAS downstream PI3K/AKT/mTOR pathway *in vitro*) (Fig. 2C). However, we found the C118D substitution did abolish the increased level of mitochondrial ROS caused by the KRAS^G12V^ mutation (Fig. 2D). This discovery, coupled with the understanding that an elevated production of mitochondrial ROS is essential for KRAS mutant driven tumorigenesis [16], suggests that C118 oxidation mediates the inhibition of mutant KRAS action on mitochondrial ROS production.

We also substituted C118 with serine in KRAS to render KRAS redox‐insensitive. Serine possesses a similar structure to cysteine but is not susceptible to oxidation by cellular ROS, thus a serine substitution inhibits any post‐translational redox‐modifications (Fig. 5B) [53]. We found, like others before us, that the C118S substitution renders RAS redox‐insensitive without affecting its biochemical functions (Fig. 1) [18, 21, 22, 28, 29, 30, 31, 32, 33].

Wild‐type and mutant KRAS activity can be enhanced via C118 S‐nitrosylation (a reaction at C118 with NO) (Fig. 5B) [35, 54]. Consistent with the notion that NO activates RAS, it was found that the pan‐NO synthase inhibitor L‐NAME reduces tumor growth and enhances the beneficial effect of carboplatin‐based chemotherapy in a mouse model of KRAS and TP53 mutation‐positive nonsmall cell lung carcinomas (NSCLC) [55]. We established that the C118S substitution reduces KRAS^G12V^ driven tumor growth (Fig. 4) and reduces the rate of proliferation of MEFs expressing mutant KRAS but not of those expressing wild‐type KRAS (Fig. 2A). These data suggest that KRAS^G12V^ is normally activated by a radical mediated reaction at C118, potentially through NO‐mediated C118 S‐nitrosylation. This hypothesis is supported by our observation that the inhibition of NO production through the L‐NAME treatment selectively inhibits KRAS^G12V^ only when C118 is present (Fig. 3A). Based on these findings, we favor a model in which mutant KRAS (and not wild‐type KRAS) is slightly activated under normal conditions by a reaction with free‐radical oxidants at C118, most likely NO. Nevertheless, we do not address the impact of an increased level of NO through endogenous or exogenous means on wild‐type KRAS activity which, as previously reported, can activate wild‐type KRAS via a reaction with C118 [23, 35].

Taken together, our results suggest that by introducing different redox‐modifications at C118, two opposing redox‐pathways regulate mutant KRAS (Fig. 5C). A nitric‐oxide mediated pathway activates mutant KRAS by C118 S‐nitrosylation while a H_2_O_2_‐mediated pathway inhibits mutant KRAS by introducing a C118 sulfinic acid modification. The C118S substitution renders KRAS redox‐insensitive thereby blocking both the nitric‐oxide mediated activation of mutant KRAS and the H_2_O_2_‐mediated inhibition of mutant KRAS. Additionally, the C118D substitution inhibits mutant KRAS signaling by blocking the nitric‐oxide mediated activation of mutant KRAS (through making KRAS redox‐insensitive; Fig. 1C) and by mimicking a permanent H_2_O_2_ oxidation at C118. Due to its dual function: inhibiting redox‐modifications at C118 and mimicking a permanent oxidation of KRAS by H_2_O_2_ at C118, the C118D substitution demonstrates a more potent inhibitory effect on the activity of mutant KRAS compared to the C118S substitution.

To explore the pharmacological options of KRAS inhibition via C118 oxidation, we utilized the pro‐oxidant PQ to generate superoxide [56] which is rapidly converted to H_2_O_2_ by SODs [57]. We discovered that PQ inhibits the increased rate of proliferation of MEFs expressing KRAS^G12V^ while sparing MEFs that express KRAS^G12V/C118S^, KRAS^G12V/C118D^, KRAS^WT^, KRAS^C118S^, or KRAS^C118D^ (Fig. 3A,B). Since the C118S and C118D substitutions render KRAS redox‐insensitive (Fig. 1C), these data show that ROS produced by PQ selectively inhibits mutant KRAS by reacting with C118. This ROS‐mediated inhibition of mutant KRAS arises probably from a direct oxidation of mutant KRAS at C118 since we found one cysteine in KRAS^G12V^ to be partly oxidized after a PQ exposure (Fig. 3E). Our technique to verify the cysteine redox status is inadequate to determine the specific type of oxidative cysteine modification induced by PQ. However, previous mass spectrometry data have shown the occurrence of the RAS C118 sulfinic acid oxidative modification in a fraction of human Ras exposed to oxidants [58]. Based on this finding and our own observation with the C118D substitution, which mimics the C118 sulfinic acid oxidative modification, we assume that PQ inhibits mutant KRAS by introducing a C118 sulfinic acid oxidative modification. To inhibit mutant KRAS, we used a concentration of PQ (Fig. 3A) that did not increase the global cellular ROS level (Fig. S5). These data suggest that the inhibiting effect of PQ on mutant KRAS is not attributed to an excessive increase of cellular ROS (which is deleterious to the cell) but rather is attributed to an appropriate elevation of ROS in the correct subcellular location to oxidize and inhibit mutant KRAS.

Considering C118 S‐nitrosylation shields KRAS from any radical‐mediated nucleotide exchange [22], we speculated that C118 S‐nitrosylation might also prevent the reaction of H_2_O_2_ with C118. We hypothesized that by inhibiting NO production by NOS, we could enhance the effect of ROS on KRAS by “freeing up” C118. Indeed, we found that compared to the PQ treatment alone (Fig. 3A), the combined treatment of NO‐production inhibitor L‐NAME and PQ increases the inhibitory effect of the PQ treatment on KRAS^G12V^, while having no effect on MEFs expressing KRAS^G12V/C118S^, KRAS^G12V/C118D^, KRAS^WT^, KRAS^C118S^, or KRAS^C118D^ (Fig. 3C,D). Based on this data, we think the inhibition of NO production has a dual effect: (a) inhibiting mutant KRAS activation through C118 S‐nitrosylation and (b) enabling ROS produced by PQ to inhibit mutant KRAS by “freeing up” C118, allowing ROS to fully engage its target, C118. Murphy et al. [59] demonstrated that S‐nitrosylation of critical thiols during oxidative stress shields them from irreversible oxidation. It is possible that a similar regulatory mechanism is at play in mutant KRAS: C118 S‐nitrosylation shields C118, preventing the formation of the largely irreversible oxidative modification and preserving the thiol for reversible regulation through nitrosylation and denitrosylation.

Interestingly, the extent of oncogenic mutant KRAS inhibition by the C118D substitution is more dramatic *in vivo* than it is *in vitro*. We found that, *in vivo*, the tumor burden and short survival rate of mice injected with MEFs expressing KRAS^G12V^ are almost completely abolished by the introduction of the C118D substitution (Fig. 4).

Taken together, the *in vitro* and *in vivo* observations reveal that human oncogenic mutant KRAS is inhibited through C118 oxidation, representing a novel vulnerability of mutant KRAS.

Further investigations are necessary to comprehend the timing and spatial aspect of H_2_O_2_ and NO‐mediated effects on mutant KRAS. It is conceivable that these two reactive molecules may act on the same or on different KRAS proteins, either at the same or at different stages of KRAS‐driven tumorigenesis. Cancer redox‐biology is highly intricate. To develop safe and effective therapeutic strategies against cancer which leverage cellular redox changes, it is imperative to elucidate specific redox regulation mechanisms uniquely essential for the growth and survival of cancer cells [13]. Previous results have shown that C118 is also a key target for RAS inhibition, as it was discovered that a series of compounds containing the reactive *N*‐ethylmaleimide group covalently bind RAS at C118 and block RAS activity [60].

## 5 Conclusions

One of the main drivers of human cancer is oncogenic mutant RAS. In this study, we unveiled a novel redox‐regulation mechanism of KRAS: the inhibition of oncogenic mutant KRAS through the post‐translational modification of C118 to cysteine sulfinic acid, generated via its reaction with H_2_O_2_. This discovery paves the way for exploring potential oxidation‐based anti‐KRAS treatments in humans.

## Conflict of interest

The authors declare no conflict of interest.

## Author contributions

MK‐D and CA designed experiments and research aims, analyzed data and wrote the manuscript. MK‐D, EPe, EPa, GC, ZZ, and AM performed experiments. HW, KQ, RS, IS, M‐JN, KDW, and DS assisted with the experiments. CA, PEP, K‐DW, DS, and M‐JN edited the manuscript. CA supervised the study and acquired funding.

### Peer review

The peer review history for this article is available at https://www.webofscience.com/api/gateway/wos/peer‐review/10.1002/1878‐0261.13798.

## Supporting information


**Fig. S1.** The mRNA expression level of wild‐type and mutant KRAS carrying the C118S or C118D substitution were similar to the controls.
**Fig. S2.** The C118S substitution nor the C118D substitution interfere with the inhibiting effect of KRAS G12D specific inhibitors and with the inhibiting effect of KRAS G12C specific inhibitors.
**Fig. S3.** Under prolonged 5‐day serum‐starvation conditions, the inhibition of mutant KRAS^G12V^‐driven cell growth by the C118S substitution, and particularly by the C118D substitution, becomes more evidently pronounced.
**Fig. S4.** Analysis of the synergistic effect of the inhibitors dactolisib (PI3Ki) and trametinib (ERKi), alone or in combination, showed that KRas^lox^ KRAS^MUT^ cells expressing KRAS^G12V^ are more sensitive to the treatment than KRAS^G12D^ and KRAS^G12C^ mutant cell lines.
**Fig. S5.** Treatment with 35 μm PQ only minimally increases the ROS level to inhibit mutant KRAS, and does not increase the cellular ROS level to a cytotoxic level.
**Fig. S6.** Human cell lines harboring a KRAS^G12V^ mutation was strongly inhibited by PQ treatment, whereas cells harboring KRAS^G12C^ or KRAS^G12D^ mutations were only weakly affected.
**Fig. S7.** NAC increases the growth of MEFs expressing KRAS^G12V^.
**Fig. S8.** Analysis of RAS effector activation in *ex‐vivo* tumor samples (KRas^lox^ KRAS^MUT^ MEFs expressing KRAS^G12V^, KRAS^G12V/C118S^, and KRAS^G12V/C118D^) taken at the end point of the survival experiment.
**Fig. S9.** X‐ray KRAS structure model, C118 is part of the RAS–RAS interaction interface through a water network.

**Table S1.** Key resources table.

## Data Availability

Data from this study are available from the corresponding author upon reasonable request.
