# Supplementary material for: Oncogenic mutant KRAS inhibition through oxidation at cysteine 118
Source: Mol Oncol. 2025 Jan 21;19(2):311–28. doi: 10.1002/1878-0261.13798 (PMC11793020; doi:10.1002/1878-0261.13798)
Supplement: Supplementary file 1 — Fig. S1. The mRNA expression level of wild‐type and mutant KRAS carrying the C118S or C118D substitution were similar to the controls. Fig. S2. The C118S substitution nor the C118D substitution interfere with the inhibiting effect of KRAS G12D specific inhibitors and with the inhibiting effect of KRAS G12C specific inhibitors. Fig. S3. Under prolonged 5‐day serum‐starvation conditions, the inhibition of mutant KRASG12V‐driven cell growth by the C118S substitution, and particularly by the C118D substitution, becomes more evidently pronounced. Fig. S4. Analysis of the synergistic effect of the inhibitors dactolisib (PI3Ki) and trametinib (ERKi), alone or in combination, showed that KRaslox KRASMUT cells expressing KRASG12V are more sensitive to the treatment than KRASG12D and KRASG12C mutant cell lines. Fig. S5. Treatment with 35 μm PQ only minimally increases the ROS level to inhibit mutant KRAS, and does not increase the cellular ROS level to a cytotoxic level. Fig. S6. Human cell lines harboring a KRASG12V mutation was strongly inhibited by PQ treatment, whereas cells harboring KRASG12C or KRASG12D mutations were only weakly affected. Fig. S7. NAC increases the growth of MEFs expressing KRASG12V. Fig. S8. Analysis of RAS effector activation in ex‐vivo tumor samples (KRaslox KRASMUT MEFs expressing KRASG12V, KRASG12V/C118S, and KRASG12V/C118D) taken at the end point of the survival experiment. Fig. S9. X‐ray KRAS structure model, C118 is part of the RAS–RAS interaction interface through a water network. [file MOL2-19-311-s001.zip › Supplementary Legends.docx]

**Supplementary Figure 1.** Real-time PCR quantification of the relative expression of KRAS in KRas^lox^ KRAS^MUT^ Mouse Embryonic Fibroblasts (MEFs) expressing KRAS^WT^, KRAS^G12C^, KRAS^G12D^, or KRAS^G12V^ with or without the in *cis* C118D substitution.

**Supplementary Figure 2**. A) Growth rates of KRas^lox^ KRAS^MUT^ Mouse Embryonic Fibroblasts (MEFs) expressing KRAS^G12D^, KRAS^G12D/C118S^ and KRAS^G12D/C118D^ treated or not treated with 1uM MRTX1133 (a selective G12D inhibitor) or the negative control AMG510 (a selective G12C inhibitor) assessed by IncuCyte measurements. B) Growth rates of KRas^lox^ KRAS^MUT^ MEFs expressing KRAS^G12C^, KRAS^G12C/C118S^ and KRAS^G12C/C118D^ treated or not treated with 1uM AMG510 (a selective G12C inhibitor) and MRTX849 (a selective G12C inhibitor) or the negative control MRTX1133 (a selective G12D inhibitor) assessed by IncuCyte measurements.

**Supplementary Figure 3**.Growth rates of in KRas^lox^ KRAS^MUT^ Mouse Embryonic Fibroblasts (MEFs) expressing KRAS^WT^ or KRAS^G12V^ with or without the in *cis* C118S or C118D substitution grown in 0.5% fetal bovine serum (FBS) medium assessed by a prolonged 5- days IncuCyte measurements.

**Supplementary Figure 4.** Analysis of synergistic effect of dactolisib and trametinib in combination on KRas^lox^ KRAS^MUT^ Mouse Embryonic Fibroblasts (MEFs) expressing KRAS^G12V^, KRAS^G12C^ or KRAS^G12D^ with or without the in cis C118S or C118D substitution.

**Supplementary Figure 5**. Mean cellular Reactive Oxygen Species (ROS) levels expressed in % relative to control detected with H_2_DCFDA (which oxidizes to 2’-7’dichlorofluorescein (DCF)) in KRas^lox^ KRAS^MUT^ Mouse Embryonic Fibroblasts (MEFs) expressing KRAS^WT^ or KRAS^G12V^ treated or not with 35uM or 500uM PQ.

**Supplementary Figure 6**. Growth rates of human cell line with KRAS ^G12V^ (H441, H2887), KRAS^G12C^ (H23, H358), or KRAS^G12D^ (A427, SK-LU-1) mutation treated or not treated with 17,5 or 35 uM PQ assessed by IncuCyte measurements.

**Supplementary Figure 7**. Growth rates KRas^lox^ KRAS^MUT^ Mouse Embryonic Fibroblasts (MEFs) expressing KRAS^G12V^, KRAS^G12V/C118S^ and KRAS^G12V/C118D^ treated or not treated with 4mM NAC alone, in combination with 35uM PQ and 1mM L-NAME or in combination with 35uM PQ, 1mM L-NAME and 4mM NAC assessed by IncuCyte measurements.

**Supplementary Figure 8.** Analysis of RAS effector activation in *ex-vivo* tumor samples (three tumors per cell lines KRas^lox^ KRAS^MUT^ MEFs expressing KRAS^G12V^, KRAS^G12V/C118S^ and KRAS^G12V/C118D^) taken at the end point of the survival experiment, with densitometry analysis of Western blots of KRAS^G12V^, KRAS^G12V/C118S^ and KRAS^G12V/C118D^ tumors where ERK activity is represented as pERK/tERK ratio and AKT activity represented as pAKT/tAKT ratio.

**Supplementary Figure 9.** Crystal structure of human WT-KRAS in complex with GDP (PDB 5w22) shows KRAS C118S engaged in a symmetric interaction around the α4-α5 interface. The PyMOL Molecular Graphics System, Version 3.0.4, Schrödinger, LLC has been used to generate this model.
