# Supplementary figures and images for: Oncogenic mutant KRAS inhibition through oxidation at cysteine 118
Source: Mol Oncol. 2025 Jan 21;19(2):311–28. doi: 10.1002/1878-0261.13798 (PMC11793020; doi:10.1002/1878-0261.13798)

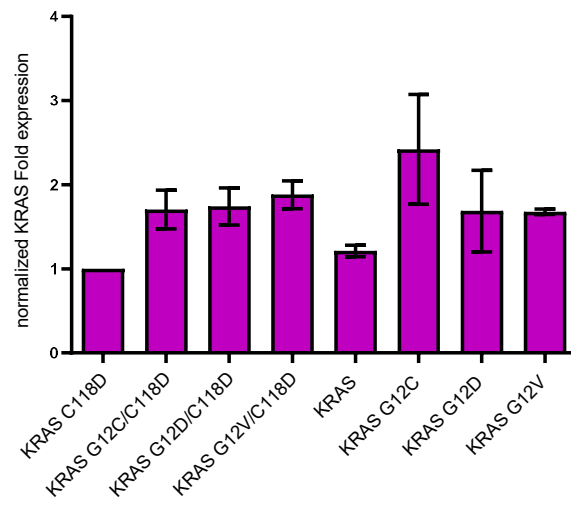

**Supplemental 1**

Supplement: Supplementary file 1 — Fig. S1. The mRNA expression level of wild‐type and mutant KRAS carrying the C118S or C118D substitution were similar to the controls. Fig. S2. The C118S substitution nor the C118D substitution interfere with the inhibiting effect of KRAS G12D specific inhibitors and with the inhibiting effect of KRAS G12C specific inhibitors. Fig. S3. Under prolonged 5‐day serum‐starvation conditions, the inhibition of mutant KRASG12V‐driven cell growth by the C118S substitution, and particularly by the C118D substitution, becomes more evidently pronounced. Fig. S4. Analysis of the synergistic effect of the inhibitors dactolisib (PI3Ki) and trametinib (ERKi), alone or in combination, showed that KRaslox KRASMUT cells expressing KRASG12V are more sensitive to the treatment than KRASG12D and KRASG12C mutant cell lines. Fig. S5. Treatment with 35 μm PQ only minimally increases the ROS level to inhibit mutant KRAS, and does not increase the cellular ROS level to a cytotoxic level. Fig. S6. Human cell lines harboring a KRASG12V mutation was strongly inhibited by PQ treatment, whereas cells harboring KRASG12C or KRASG12D mutations were only weakly affected. Fig. S7. NAC increases the growth of MEFs expressing KRASG12V. Fig. S8. Analysis of RAS effector activation in ex‐vivo tumor samples (KRaslox KRASMUT MEFs expressing KRASG12V, KRASG12V/C118S, and KRASG12V/C118D) taken at the end point of the survival experiment. Fig. S9. X‐ray KRAS structure model, C118 is part of the RAS–RAS interaction interface through a water network. [file MOL2-19-311-s001.zip › mol213798-sup-0001-FigureS1.pdf]

A

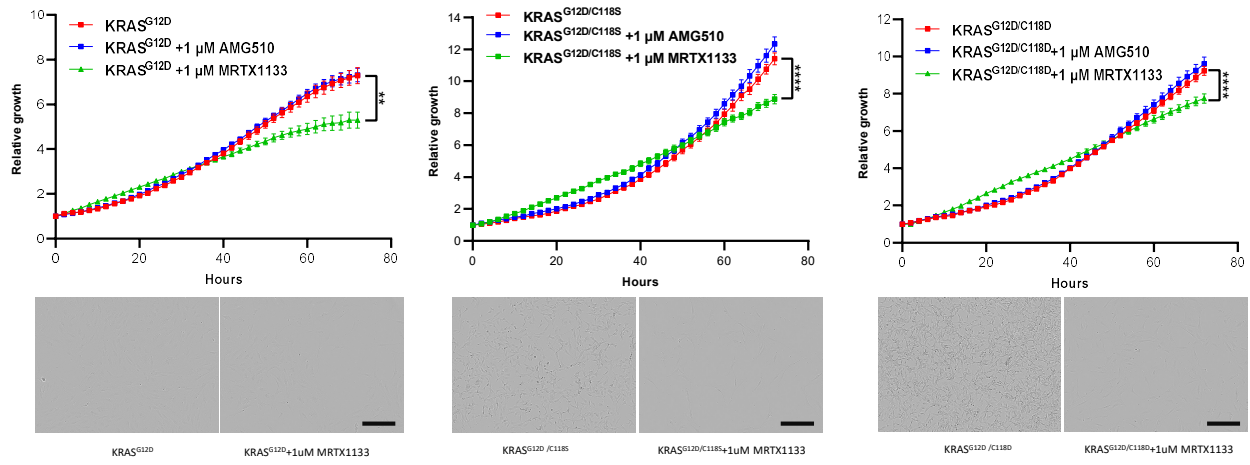

B

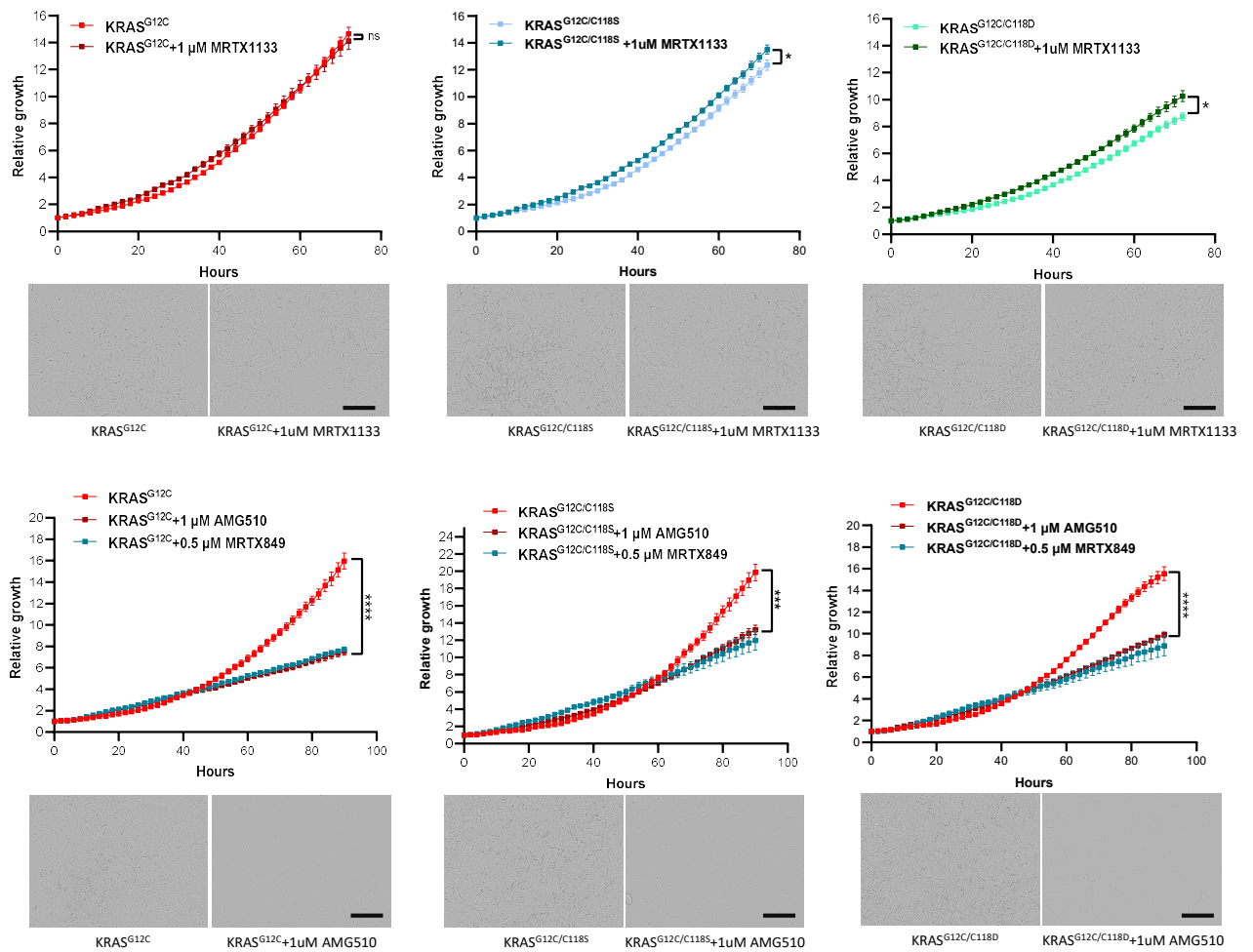

Supplement: Supplementary file 1 — Fig. S1. The mRNA expression level of wild‐type and mutant KRAS carrying the C118S or C118D substitution were similar to the controls. Fig. S2. The C118S substitution nor the C118D substitution interfere with the inhibiting effect of KRAS G12D specific inhibitors and with the inhibiting effect of KRAS G12C specific inhibitors. Fig. S3. Under prolonged 5‐day serum‐starvation conditions, the inhibition of mutant KRASG12V‐driven cell growth by the C118S substitution, and particularly by the C118D substitution, becomes more evidently pronounced. Fig. S4. Analysis of the synergistic effect of the inhibitors dactolisib (PI3Ki) and trametinib (ERKi), alone or in combination, showed that KRaslox KRASMUT cells expressing KRASG12V are more sensitive to the treatment than KRASG12D and KRASG12C mutant cell lines. Fig. S5. Treatment with 35 μm PQ only minimally increases the ROS level to inhibit mutant KRAS, and does not increase the cellular ROS level to a cytotoxic level. Fig. S6. Human cell lines harboring a KRASG12V mutation was strongly inhibited by PQ treatment, whereas cells harboring KRASG12C or KRASG12D mutations were only weakly affected. Fig. S7. NAC increases the growth of MEFs expressing KRASG12V. Fig. S8. Analysis of RAS effector activation in ex‐vivo tumor samples (KRaslox KRASMUT MEFs expressing KRASG12V, KRASG12V/C118S, and KRASG12V/C118D) taken at the end point of the survival experiment. Fig. S9. X‐ray KRAS structure model, C118 is part of the RAS–RAS interaction interface through a water network. [file MOL2-19-311-s001.zip › mol213798-sup-0002-FigureS2.pdf]

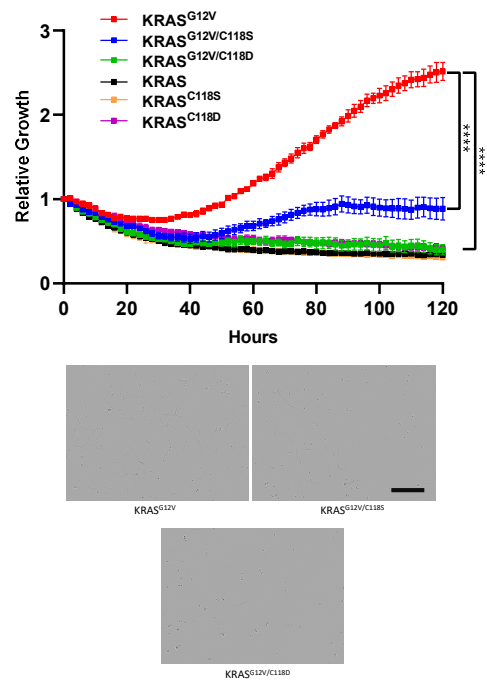

Supplemental 3

Supplement: Supplementary file 1 — Fig. S1. The mRNA expression level of wild‐type and mutant KRAS carrying the C118S or C118D substitution were similar to the controls. Fig. S2. The C118S substitution nor the C118D substitution interfere with the inhibiting effect of KRAS G12D specific inhibitors and with the inhibiting effect of KRAS G12C specific inhibitors. Fig. S3. Under prolonged 5‐day serum‐starvation conditions, the inhibition of mutant KRASG12V‐driven cell growth by the C118S substitution, and particularly by the C118D substitution, becomes more evidently pronounced. Fig. S4. Analysis of the synergistic effect of the inhibitors dactolisib (PI3Ki) and trametinib (ERKi), alone or in combination, showed that KRaslox KRASMUT cells expressing KRASG12V are more sensitive to the treatment than KRASG12D and KRASG12C mutant cell lines. Fig. S5. Treatment with 35 μm PQ only minimally increases the ROS level to inhibit mutant KRAS, and does not increase the cellular ROS level to a cytotoxic level. Fig. S6. Human cell lines harboring a KRASG12V mutation was strongly inhibited by PQ treatment, whereas cells harboring KRASG12C or KRASG12D mutations were only weakly affected. Fig. S7. NAC increases the growth of MEFs expressing KRASG12V. Fig. S8. Analysis of RAS effector activation in ex‐vivo tumor samples (KRaslox KRASMUT MEFs expressing KRASG12V, KRASG12V/C118S, and KRASG12V/C118D) taken at the end point of the survival experiment. Fig. S9. X‐ray KRAS structure model, C118 is part of the RAS–RAS interaction interface through a water network. [file MOL2-19-311-s001.zip › mol213798-sup-0003-FigureS3.pdf]

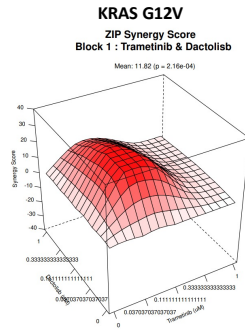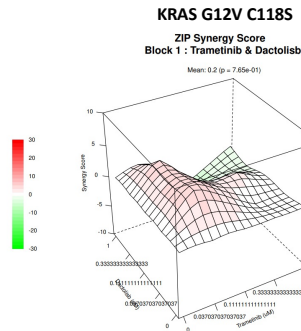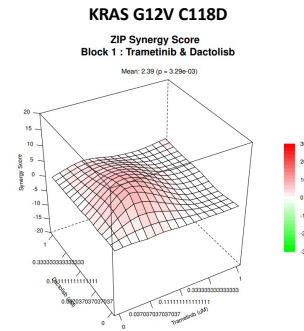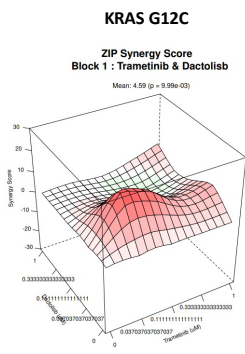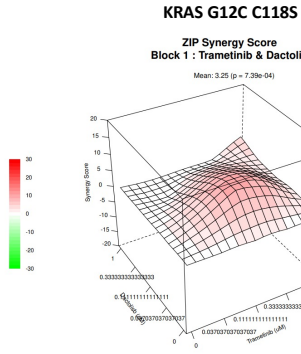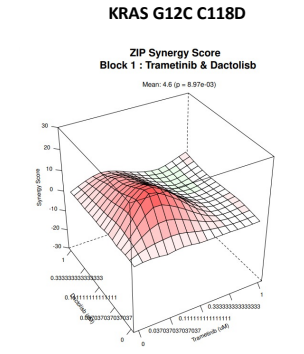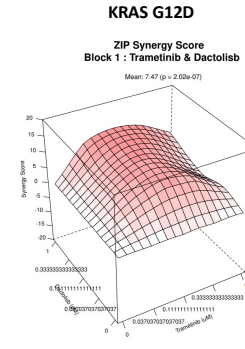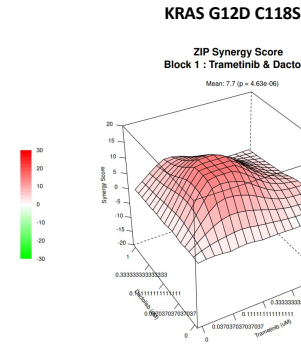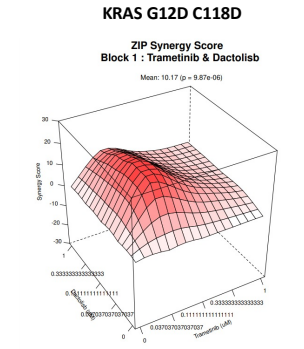

Supplemental 4

Supplement: Supplementary file 1 — Fig. S1. The mRNA expression level of wild‐type and mutant KRAS carrying the C118S or C118D substitution were similar to the controls. Fig. S2. The C118S substitution nor the C118D substitution interfere with the inhibiting effect of KRAS G12D specific inhibitors and with the inhibiting effect of KRAS G12C specific inhibitors. Fig. S3. Under prolonged 5‐day serum‐starvation conditions, the inhibition of mutant KRASG12V‐driven cell growth by the C118S substitution, and particularly by the C118D substitution, becomes more evidently pronounced. Fig. S4. Analysis of the synergistic effect of the inhibitors dactolisib (PI3Ki) and trametinib (ERKi), alone or in combination, showed that KRaslox KRASMUT cells expressing KRASG12V are more sensitive to the treatment than KRASG12D and KRASG12C mutant cell lines. Fig. S5. Treatment with 35 μm PQ only minimally increases the ROS level to inhibit mutant KRAS, and does not increase the cellular ROS level to a cytotoxic level. Fig. S6. Human cell lines harboring a KRASG12V mutation was strongly inhibited by PQ treatment, whereas cells harboring KRASG12C or KRASG12D mutations were only weakly affected. Fig. S7. NAC increases the growth of MEFs expressing KRASG12V. Fig. S8. Analysis of RAS effector activation in ex‐vivo tumor samples (KRaslox KRASMUT MEFs expressing KRASG12V, KRASG12V/C118S, and KRASG12V/C118D) taken at the end point of the survival experiment. Fig. S9. X‐ray KRAS structure model, C118 is part of the RAS–RAS interaction interface through a water network. [file MOL2-19-311-s001.zip › mol213798-sup-0004-FigureS4.pdf]

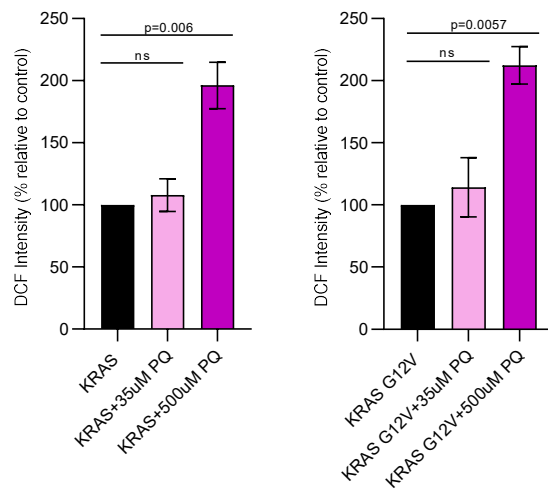

**Supplemental 5**

Supplement: Supplementary file 1 — Fig. S1. The mRNA expression level of wild‐type and mutant KRAS carrying the C118S or C118D substitution were similar to the controls. Fig. S2. The C118S substitution nor the C118D substitution interfere with the inhibiting effect of KRAS G12D specific inhibitors and with the inhibiting effect of KRAS G12C specific inhibitors. Fig. S3. Under prolonged 5‐day serum‐starvation conditions, the inhibition of mutant KRASG12V‐driven cell growth by the C118S substitution, and particularly by the C118D substitution, becomes more evidently pronounced. Fig. S4. Analysis of the synergistic effect of the inhibitors dactolisib (PI3Ki) and trametinib (ERKi), alone or in combination, showed that KRaslox KRASMUT cells expressing KRASG12V are more sensitive to the treatment than KRASG12D and KRASG12C mutant cell lines. Fig. S5. Treatment with 35 μm PQ only minimally increases the ROS level to inhibit mutant KRAS, and does not increase the cellular ROS level to a cytotoxic level. Fig. S6. Human cell lines harboring a KRASG12V mutation was strongly inhibited by PQ treatment, whereas cells harboring KRASG12C or KRASG12D mutations were only weakly affected. Fig. S7. NAC increases the growth of MEFs expressing KRASG12V. Fig. S8. Analysis of RAS effector activation in ex‐vivo tumor samples (KRaslox KRASMUT MEFs expressing KRASG12V, KRASG12V/C118S, and KRASG12V/C118D) taken at the end point of the survival experiment. Fig. S9. X‐ray KRAS structure model, C118 is part of the RAS–RAS interaction interface through a water network. [file MOL2-19-311-s001.zip › mol213798-sup-0005-FigureS5.pdf]

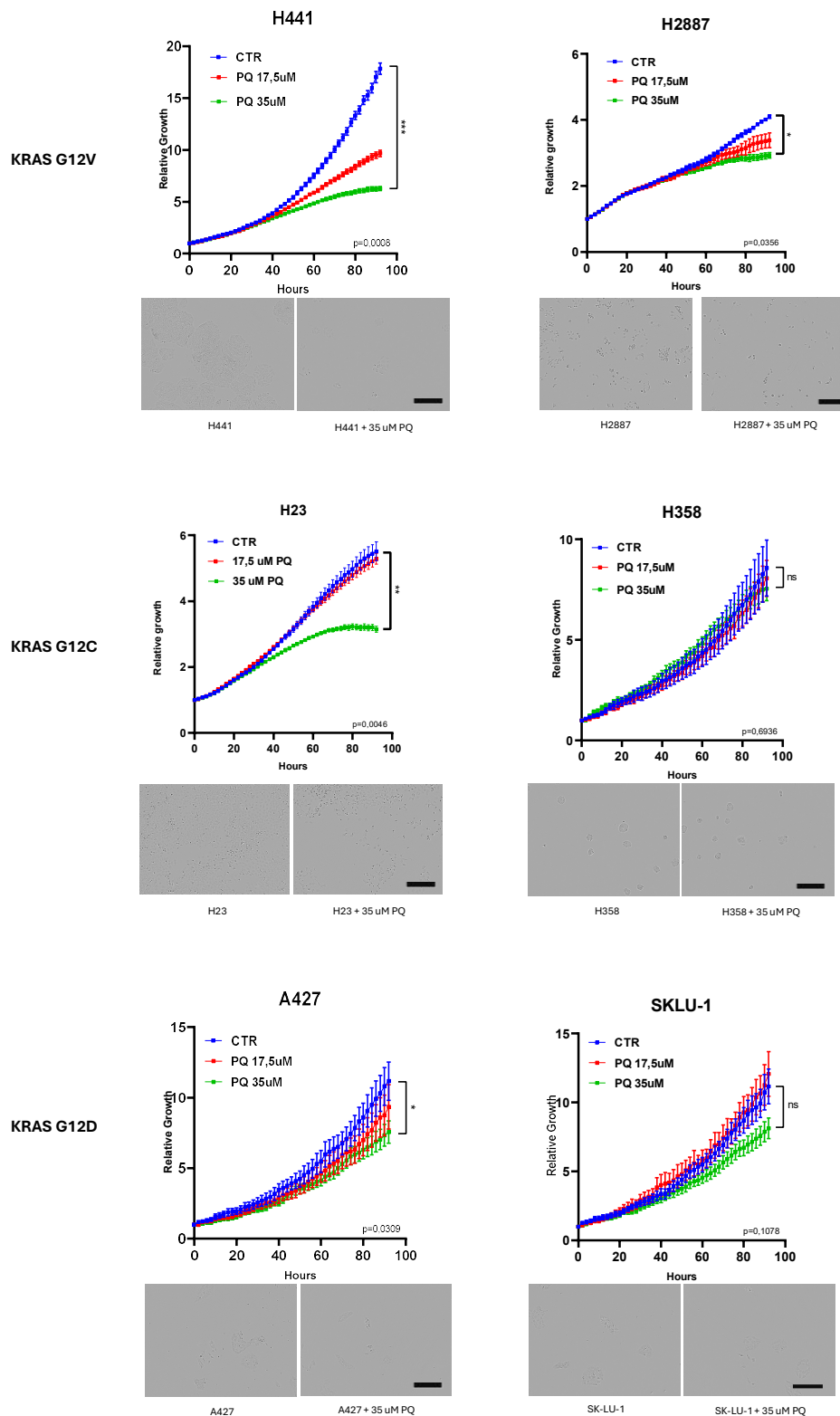

Supplement: Supplementary file 1 — Fig. S1. The mRNA expression level of wild‐type and mutant KRAS carrying the C118S or C118D substitution were similar to the controls. Fig. S2. The C118S substitution nor the C118D substitution interfere with the inhibiting effect of KRAS G12D specific inhibitors and with the inhibiting effect of KRAS G12C specific inhibitors. Fig. S3. Under prolonged 5‐day serum‐starvation conditions, the inhibition of mutant KRASG12V‐driven cell growth by the C118S substitution, and particularly by the C118D substitution, becomes more evidently pronounced. Fig. S4. Analysis of the synergistic effect of the inhibitors dactolisib (PI3Ki) and trametinib (ERKi), alone or in combination, showed that KRaslox KRASMUT cells expressing KRASG12V are more sensitive to the treatment than KRASG12D and KRASG12C mutant cell lines. Fig. S5. Treatment with 35 μm PQ only minimally increases the ROS level to inhibit mutant KRAS, and does not increase the cellular ROS level to a cytotoxic level. Fig. S6. Human cell lines harboring a KRASG12V mutation was strongly inhibited by PQ treatment, whereas cells harboring KRASG12C or KRASG12D mutations were only weakly affected. Fig. S7. NAC increases the growth of MEFs expressing KRASG12V. Fig. S8. Analysis of RAS effector activation in ex‐vivo tumor samples (KRaslox KRASMUT MEFs expressing KRASG12V, KRASG12V/C118S, and KRASG12V/C118D) taken at the end point of the survival experiment. Fig. S9. X‐ray KRAS structure model, C118 is part of the RAS–RAS interaction interface through a water network. [file MOL2-19-311-s001.zip › mol213798-sup-0006-FigureS6.pdf]

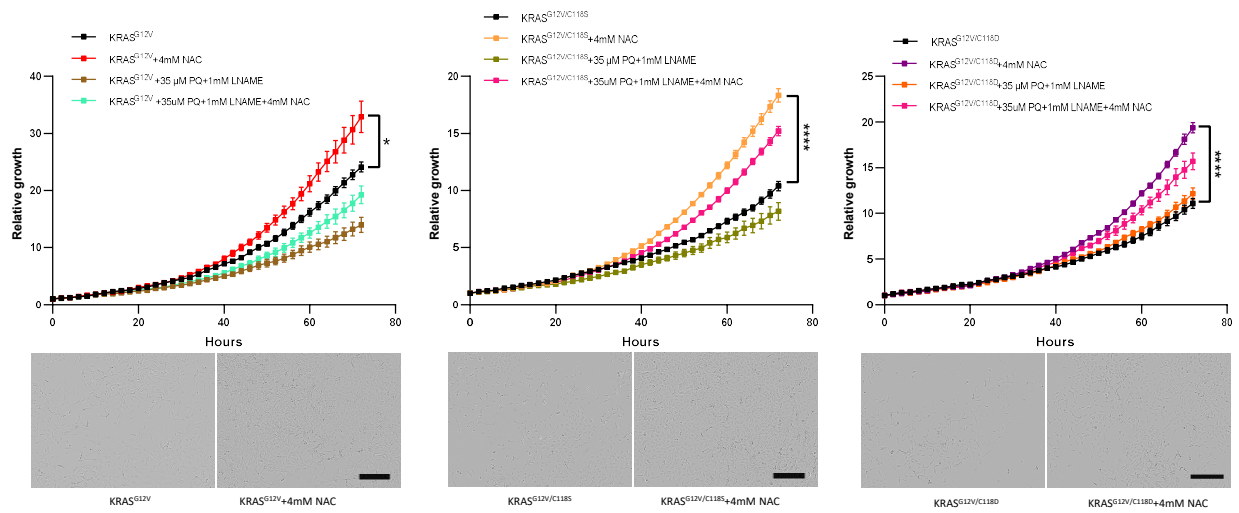

Supplemental 7

Supplement: Supplementary file 1 — Fig. S1. The mRNA expression level of wild‐type and mutant KRAS carrying the C118S or C118D substitution were similar to the controls. Fig. S2. The C118S substitution nor the C118D substitution interfere with the inhibiting effect of KRAS G12D specific inhibitors and with the inhibiting effect of KRAS G12C specific inhibitors. Fig. S3. Under prolonged 5‐day serum‐starvation conditions, the inhibition of mutant KRASG12V‐driven cell growth by the C118S substitution, and particularly by the C118D substitution, becomes more evidently pronounced. Fig. S4. Analysis of the synergistic effect of the inhibitors dactolisib (PI3Ki) and trametinib (ERKi), alone or in combination, showed that KRaslox KRASMUT cells expressing KRASG12V are more sensitive to the treatment than KRASG12D and KRASG12C mutant cell lines. Fig. S5. Treatment with 35 μm PQ only minimally increases the ROS level to inhibit mutant KRAS, and does not increase the cellular ROS level to a cytotoxic level. Fig. S6. Human cell lines harboring a KRASG12V mutation was strongly inhibited by PQ treatment, whereas cells harboring KRASG12C or KRASG12D mutations were only weakly affected. Fig. S7. NAC increases the growth of MEFs expressing KRASG12V. Fig. S8. Analysis of RAS effector activation in ex‐vivo tumor samples (KRaslox KRASMUT MEFs expressing KRASG12V, KRASG12V/C118S, and KRASG12V/C118D) taken at the end point of the survival experiment. Fig. S9. X‐ray KRAS structure model, C118 is part of the RAS–RAS interaction interface through a water network. [file MOL2-19-311-s001.zip › mol213798-sup-0007-FigureS7.pdf]

A

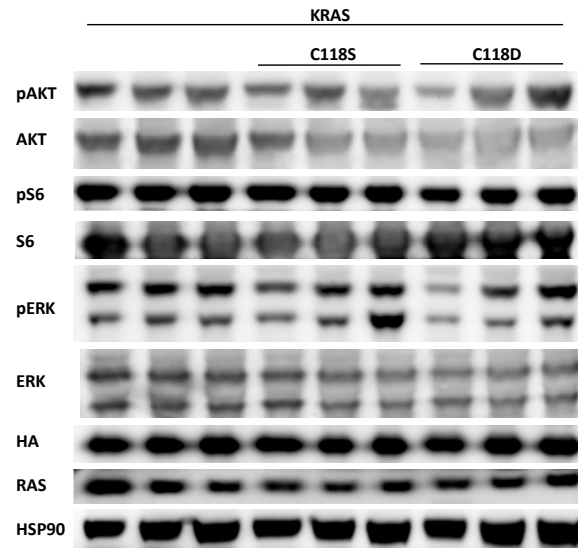

B

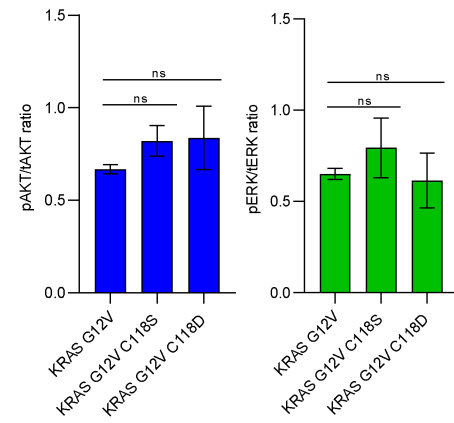

Supplemental 8

Supplement: Supplementary file 1 — Fig. S1. The mRNA expression level of wild‐type and mutant KRAS carrying the C118S or C118D substitution were similar to the controls. Fig. S2. The C118S substitution nor the C118D substitution interfere with the inhibiting effect of KRAS G12D specific inhibitors and with the inhibiting effect of KRAS G12C specific inhibitors. Fig. S3. Under prolonged 5‐day serum‐starvation conditions, the inhibition of mutant KRASG12V‐driven cell growth by the C118S substitution, and particularly by the C118D substitution, becomes more evidently pronounced. Fig. S4. Analysis of the synergistic effect of the inhibitors dactolisib (PI3Ki) and trametinib (ERKi), alone or in combination, showed that KRaslox KRASMUT cells expressing KRASG12V are more sensitive to the treatment than KRASG12D and KRASG12C mutant cell lines. Fig. S5. Treatment with 35 μm PQ only minimally increases the ROS level to inhibit mutant KRAS, and does not increase the cellular ROS level to a cytotoxic level. Fig. S6. Human cell lines harboring a KRASG12V mutation was strongly inhibited by PQ treatment, whereas cells harboring KRASG12C or KRASG12D mutations were only weakly affected. Fig. S7. NAC increases the growth of MEFs expressing KRASG12V. Fig. S8. Analysis of RAS effector activation in ex‐vivo tumor samples (KRaslox KRASMUT MEFs expressing KRASG12V, KRASG12V/C118S, and KRASG12V/C118D) taken at the end point of the survival experiment. Fig. S9. X‐ray KRAS structure model, C118 is part of the RAS–RAS interaction interface through a water network. [file MOL2-19-311-s001.zip › mol213798-sup-0008-FigureS8.pdf]

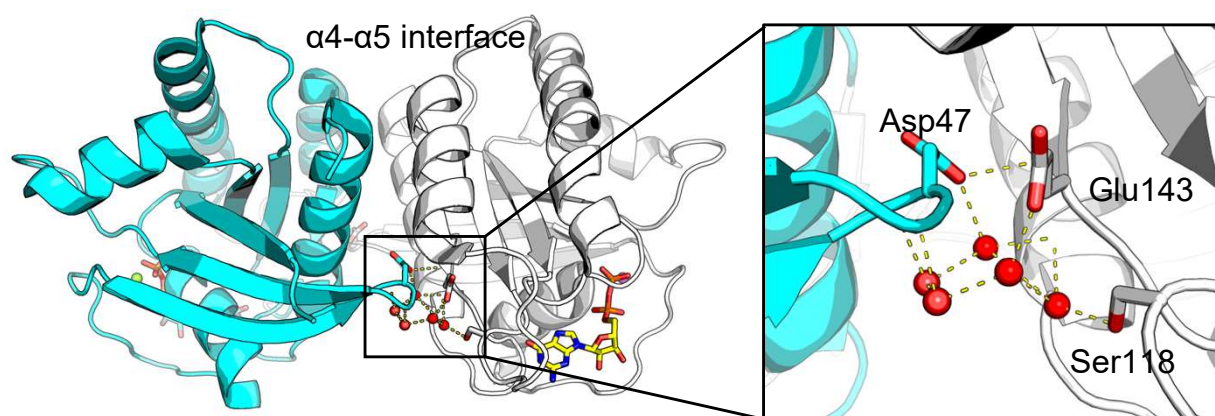

Supplemental 9

Supplement: Supplementary file 1 — Fig. S1. The mRNA expression level of wild‐type and mutant KRAS carrying the C118S or C118D substitution were similar to the controls. Fig. S2. The C118S substitution nor the C118D substitution interfere with the inhibiting effect of KRAS G12D specific inhibitors and with the inhibiting effect of KRAS G12C specific inhibitors. Fig. S3. Under prolonged 5‐day serum‐starvation conditions, the inhibition of mutant KRASG12V‐driven cell growth by the C118S substitution, and particularly by the C118D substitution, becomes more evidently pronounced. Fig. S4. Analysis of the synergistic effect of the inhibitors dactolisib (PI3Ki) and trametinib (ERKi), alone or in combination, showed that KRaslox KRASMUT cells expressing KRASG12V are more sensitive to the treatment than KRASG12D and KRASG12C mutant cell lines. Fig. S5. Treatment with 35 μm PQ only minimally increases the ROS level to inhibit mutant KRAS, and does not increase the cellular ROS level to a cytotoxic level. Fig. S6. Human cell lines harboring a KRASG12V mutation was strongly inhibited by PQ treatment, whereas cells harboring KRASG12C or KRASG12D mutations were only weakly affected. Fig. S7. NAC increases the growth of MEFs expressing KRASG12V. Fig. S8. Analysis of RAS effector activation in ex‐vivo tumor samples (KRaslox KRASMUT MEFs expressing KRASG12V, KRASG12V/C118S, and KRASG12V/C118D) taken at the end point of the survival experiment. Fig. S9. X‐ray KRAS structure model, C118 is part of the RAS–RAS interaction interface through a water network. [file MOL2-19-311-s001.zip › mol213798-sup-0009-FigureS9.pdf]
