# Supplementary material for: Oncogenic mutant KRAS inhibition through oxidation at cysteine 118
Source: Mol Oncol. 2025 Jan 21;19(2):311–28. doi: 10.1002/1878-0261.13798 (PMC11793020; doi:10.1002/1878-0261.13798)
Supplement: Supplementary file 2 — Table S1. Key resources table. [file MOL2-19-311-s002.docx]

# Supplementary Table S1

KEY RESOURCES TABLE

| **REAGENT or RESOURCE** | **SOURCE** | **IDENTIFIER** |
| --- | --- | --- |
| **Antibodies** |  |  |
| Mouse monoclonal, Ras Mouse mAb | Cell Signaling | Active Ras Detection Kit #8821 |
| Mouse monoclonal, Anti-HA (6E2) | Cell Signaling | Cat# 2367, RRID:AB_10691311 |
| Rabbit polyclonal, HSP90 Antibody | Cell Signaling | Cat# 4874, RRID:AB_2121214 |
| Rabbit monoclonal, Anti-phosphorylated Akt (Ser473) | Cell Signaling | Cat#4060, RRID:AB_2315049 |
| Rabbit polyclonal, p44/42 MAP kinase (phosphorylated Erk1/2) | Cell Signaling | Cat# 9101, RRID:AB_331646 |
| Rabbit polyclonal, Phospho-S6 Ribosomal Protein | Cell Signaling | Cat# 2215, RRID:AB_331682 |
| Rabbit monoclonal, Anti-phosphorylated Akt (Ser473) | Cell Signaling | Cat#4060, RRID:AB_2315049 |
| Rabbit polyclonal, Anti-Akt | Cell Signaling | Cat#9272, RRID:AB_329827 |
| Rabbit polyclonal, p44/42 MAPK (Erk1/2) Antibody | Cell Signaling | Cat# 9102, RRID:AB_330744 |
| Rabbit monoclonal, Anti-S6 ribosomal protein | Cell Signaling | Cat#2217, RRID:AB_331355 |
| ECL Sheep anti-Mouse IgG, HRP-linked secondary antibody | GE Healthcare | Cat#NA931V, RRID:AB_772193 |
| ECL Donkey anti-Rabbit IgG, HRP-linked secondary antibody | GE Healthcare | Cat#NA934V, RRID:AB_772191 |
| **Chemicals, Peptides, and Recombinant Proteins** |  |  |
| 4OHT ((Z)-4-Hydroxytamoxifen) | Sigma-Aldrich | Cat#H7904 |
| MitoSOX^TM^ | Invitrogen | Cat#M36008 |
| 2',7'-dichlorodihydrofluorescein diacetate (DCFH-DA) | Sigma-Aldrich | Cat#D6883 |
| Methyl viologen dichloride hydrate | Sigma-Aldrich | Cat# 856177 |
| N-Acetyl-Cysteine (NAC | Sigma-Aldrich | Cat# A7250 |
| Sotorasib (Synonyms: AMG-510) | Selleck Chem | Cat# S8830 |
| MRTX1133 | Selleck Chem | Cat# E1051 |
| Adagrasib (Synonyms: MRTX849) | Selleck Chem | Cat# S8884 |
| RMC-4998 | MedChemExpress | Cat# HY-156671 |
| Nω-Nitro-L-arginine methyl ester hydrochloride (L-NAME) | Sigma-Aldrich | Cat# N5751 |
| TRIZOL isolation protocol | Thermo Fisher | Cat#15596018 |
| RIPA lysis buffer | Thermo Fisher | Cat#89900 |
| Halt protease and phosphatase inhibitor cocktail | Thermo Fisher | Cat#78445 |
| PEG-PCMAL | SulfoBiotics | Cat# SB20 |
| **REAGENT or RESOURCE** | **SOURCE** | **IDENTIFIER** |
| **Sequence based reagents** |  |  |
| Murine ActinB Fw: GGCTGTATTCCCCTCCATCG | [38] | N/A |
| Murine ActinB Rev: CCAGTTGGTAACAATGCCATGT | [38] | N/A |
| Human KRAS Fw: GGACTGGGGAGGGCTTTCT | [38] | N/A |
| Human KRAS Rev: GCCTGTTTTGTGTCTACTGTTCT | [38] | N/A |
| **Critical Commercial Assays** |  |  |
| QuikChange XL Site-Directed Mutagenesis Kit | Agilent | Cat#200516 |
| Effectene Transfection Reagent | Qiagen | Cat#301425 |
| RevertAid RT Kit | Thermo Fisher | Cat#00940535 |
| PowerTrack SYBR Green Master Mix | applied biosystems | Cat#00864923 |
| OxyBlot™ Protein Oxidation Detection Kit | Merk Millipore | Cat# S7150 |
| RAS pull-down and detection kit | Cell Signaling | Cat#11871 |
| **Experimental Models: Mouse strains** |  |  |
| 6 to 8 week-old female Nude-*Foxn1nu/Foxn1+* athymic mice | Envigo | Hsd:Athymic Nude-*Foxn1nu/Foxn1+* |
| **Experimental Models: cell lines** |  |  |
| KRas^lox^ KRAS^MUT^ MEFs | (Ambrogio et al. 2018) | N/A |
| H441; human G12V-mutant, lung cancer male | Cellosaurus | (RRID:CVCL_1561) |
| H2887; human G12V-mutant, lung cancer, male | Cellosaurus | (RRID:CVCL_5159) |
| H23; human G12C-mutant NSCLC, male | Cellosaurus | (RRID:CVCL_1547) |
| H358; human G12C-mutant NSCLC, male | Cellosaurus | (RRID:CVCL_1559) |
| A427; human G12D-mutant lung carcinoma; male | Cellosaurus | (RRID:CVCL_1055) |
| SK-LU-1; human G12D-mutant lung adenocarcinoma; female | Cellosaurus | (RRID:CVCL_0629) |
| **Recombinant DNA** |  |  |
| pBABE human KRAS WT HA-tagged | Provided by C. Der | Addgene Cat#75282 |
| pBABE human KRAS C118S HA-tagged | This paper | N/A |
| pBABE human KRAS C118D HA-tagged | This paper | N/A |
| pBABE human KRAS G12C HA-tagged | This paper | N/A |
| pBABE human KRAS G12D HA-tagged | This paper | N/A |
| pBABE human KRAS G12V HA-tagged | This paper | N/A |
| pBABE human KRAS G12C/C118S HA-tagged | This paper | N/A |
| pBABE human KRAS G12D/C118S HA-tagged | This paper | N/A |
| pBABE human KRAS G12V/C118S HA-tagged | This paper | N/A |
| pBABE human KRAS G12C HA-tagged | This paper | N/A |
| pBABE human KRAS G12D HA-tagged | This paper | N/A |
| pBABE human KRAS G12V HA-tagged | This paper | N/A |
| pBABE human KRAS G12C/C118D HA-tagged | This paper | N/A |
| pBABE human KRAS G12D/C118D HA-tagged | This paper | N/A |
| pBABE human KRAS G12V/C118D HA-tagged | This paper | N/A |
| **Softwares and Algorithms** |  |  |
| GraphPad Prism | GraphPad | https://www.graphpad.com/  scientific-software/prism/ |
| ImageJ ZEN | IMAGEJ ZEISS | https://imagej.net/Fiji |
| SynergyFinder | SynergyFinder | <https://synergyfinderplus.org> |
